# Hypernetwork Construction and Feature Fusion Analysis Based on Sparse Group Lasso Method on fMRI Dataset

**DOI:** 10.3389/fnins.2020.00060

**Published:** 2020-02-12

**Authors:** Yao Li, Chao Sun, Pengzu Li, Yunpeng Zhao, Godfred Kim Mensah, Yong Xu, Hao Guo, Junjie Chen

**Affiliations:** ^1^College of Information and Computer, Taiyuan University of Technology, Taiyuan, China; ^2^College of Arts, Taiyuan University of Technology, Taiyuan, China; ^3^Department of Psychiatry, First Hospital of Shanxi Medical University, Taiyuan, China

**Keywords:** hypernetwork, sparse group lasso, cluster coefficients based on pairs of nodes, multi-feature, classification, depression

## Abstract

Recent works have shown that the resting-state brain functional connectivity hypernetwork, where multiple nodes can be connected, are an effective technique for brain disease diagnosis and classification research. The lasso method was used to construct hypernetworks by solving sparse linear regression models in previous research. But, constructing a hypernetwork based on the lasso method simply selects a single variable, in that it lacks the ability to interpret the grouping effect. Considering the group structure problem, the previous study proposed to create a hypernetwork based on the elastic net and the group lasso methods, and the results showed that the former method had the best classification performance. However, the highly correlated variables selected by the elastic net method were not necessarily in the active set in the group. Therefore, we extended our research to address this issue. Herein, we propose a new method that introduces the sparse group lasso method to improve the construction of the hypernetwork by solving the group structure problem of the brain regions. We used the traditional lasso, group lasso method, and sparse group lasso method to construct a hypernetwork in patients with depression and normal subjects. Meanwhile, other clustering coefficients (clustering coefficients based on pairs of nodes) were also introduced to extract features with traditional clustering coefficients. Two types of features with significant differences obtained after feature selection were subjected to multi-kernel learning for feature fusion and classification using each method, respectively. The network topology results revealed differences among the three networks, where hypernetwork using the lasso method was the strictest; the group lasso, most lenient; and the sgLasso method, moderate. The network topology of the sparse group lasso method was similar to that of the group lasso method but different from the lasso method. The classification results show that the sparse group lasso method achieves the best classification accuracy by using multi-kernel learning, which indicates that better classification performance can be achieved when the group structure exists and is properly extended.

## Introduction

In recent years, neuroimaging techniques have become increasingly popular for the exploration of interactions among brain regions. Blood oxygen level-dependent (BOLD) signal as a neurophysiological indicator on resting-state functional magnetic resonance imaging (rs-fMRI) can detect spontaneous low-frequency brain activity (Zeng et al., 2012). The interaction between brain regions at rest can be denoted by a functional connectivity network constructed by BOLD signals. Complex brain network research can help elucidate the mechanisms underlying mental disorders and possibly show relevant imaging markers, which provide new perspectives for the diagnosis and evaluation of clinical brain diseases (Nixon et al., 2018). Therefore, brain function network models have been successfully used to study the diagnosis and classification of neuropsychiatric diseases, including epilepsy (Zhang et al., 2012), depression (Kaiser et al., 2016), Alzheimer’s disease (Pievani et al., 2011), and schizophrenia (Lynall et al., 2010).

According to image data obtained by fMRI, many analysis approaches have been proposed to construct functional brain connectivity network models, including correlation-based approach (Bullmore and Sporns, 2009; Sporns, 2011; Wee et al., 2012; Jie et al., 2014), graphical models (Bullmore et al., 2000; Chen and Herskovits, 2007), partial correlation approach (Salvador et al., 2005; Marrelec et al., 2006, 2007), and the sparse representation approach (Lee et al., 2011; Wee et al., 2014). Most existing studies have successfully applied the correlation-based approach to the classification of patients with depression and normal controls (Zeng et al., 2012; Ye et al., 2015; Monti and Hyv Rinen, 2018); however, this approach can only capture pairs of relationships without effectively expressing interactions among multiple brain regions (Huang et al., 2010). In addition, there are many false connections given the arbitrary selection of thresholds based on the correlation network (Wee et al., 2014). Graphical models are limited in that they are confirmative, rather than exploratory, so it makes them inadequate for measuring brain functional connectivity, because little prior knowledge is adopted in studying brain functional connectivity (Huang et al., 2010). One of the algorithms based on partial correlation network models–the sparse inverse covariance matrix (SICE) estimating the magnitude of connectivity–is not appropriate because of the shrinking effect, and it is very sensitive to the regularization parameters (Wee et al., 2014). Sparse representations have also been proposed for building functional connectivity networks (Lee et al., 2011). Wee et al. used the group lasso method based on l_2,1_ regularized for building functional connectivity networks to classify normal subjects and patients to estimate using the same topology but connection networks with different connection strengths (Wee et al., 2014). Yet, the network topology mode of a particular group is ignored.

Most of the above-mentioned methods describe the relationship between two brain regions. However, recent research has provided evidence for interactions among multiple regions, except for the direct relationship between two brain regions. The latest neuroscience analyses have shown necessary higher-order interactions in neuronal spiking, local field potentials, and cortical activity (Montani et al., 2009; Ohiorhenuan et al., 2010; Santos et al., 2010; Yu et al., 2011). Yu et al. (2011) compared the properties of firing patterns among local clusters of neurons (300-mm apart) with those of neurons separated by larger distances (600–2, 500 mm) and reported that the local firing patterns are distinctive; to elaborate, multi-neuronal firing patterns at larger distances can be predicted by pairwise interactions, while patterns within local clusters often show evidence of higher-order correlations. Montani et al. (2009) simulated the effects of higher-order interactions on the amount of somatosensory information transmitted by synchronous discharge rates. Santos et al. (2010) stated that the recording activity of units in paired interactions does not describe well the pattern of neuronal activity. Moreover, a previous study also indicated that a single brain region will interact directly with a few other brain regions (Huang et al., 2010). Therefore, pairwise relationships may not be accurate in discovering the higher-order information on brain network, while it may be essential for studying the pathological basis of potential neuropsychiatric diseases.

Considering the limitations of the traditional functional network approaches, several novel methods have been proposed, and the hypernetwork model is one such example (Jie et al., 2016). The above shortcomings of the conventional method can be solved by appropriately constructing a hypernetwork. The hypernetwork is based on the hypergraph theory that is a continuation of the graph, where one edge (hyperedge) can connect multiple nodes (Zhou et al., 2006). In neuroimaging, each node in a hypernetwork refers to a brain region, and each hyperedge comprises multiple nodes to represent the relationship among multiple brain regions. In past years, hypergraphs have been successfully applied in a variety of medical imaging fields, including image segmentation (Dong et al., 2015) and classification (Gao et al., 2015; Liu et al., 2016). Some recent studies have provided associations between neuroscience and hypergraphs (Davison et al., 2015; Gao et al., 2015; Jie et al., 2016; Huang et al., 2018). For example, Gao et al. used hypergraphs to combine multimodal neuroimaging information to identify MCI (mild cognitive impairment) subjects (Gao et al., 2015). Davison et al. found the existence driven by significant co-evolution within groups of functional interactions in strength over time rather than dyadic (region-to-region) information (Davison et al., 2015). Jie et al. constructed a hyper-connectivity network of brain functions by using a sparse representation method (Jie et al., 2016). Wang et al. mined network phenotype between genetic risk factors and disease status by a novel diagnosis-aligned multi-modality regression method, in which network connectivity information was represented using a hypernetwork based on sparse representation (Huang et al., 2018). Gu et al. reported a hypergraph representation method using BOLD rs-fMRI data, which divided the hyperedge into three different categories, namely bridges, stars, and clusters, to represent the binary, focus, and spatial distribution of architecture, respectively (Gu et al., 2017). Further, a novel hypergraph learning-based approach has recently been proposed to represent complex connectivity patterns in multiple brain regions (Zu et al., 2018). The remaining interesting hypergraph applications have also been found in protein function prediction and pattern recognition (Ren et al., 2011; Gallagher and Goldberg, 2013).

In a recent study, Jie et al. (2016) created a brain function hypernetwork to diagnose brain diseases. According to the rs-fMRI time series, the lasso method was used to solve the sparse linear regression model to create a hypernetwork. By using a sparse linear model, one region can be represented as a linear combination of other regions, which represents the interaction between that particular region and other regions, as well as forcing the meaningless or false interaction to be zero. However, the limitation of using the lasso method is that when selecting a specific brain region, if there is a strong correlation among other brain regions in the construction of hyperedges, the specific brain region often arbitrarily selects one of a group of brain regions in which the group structure exists (Zou and Trevor, 2005). However, studies have shown that the natural group structures are very often among brain regions, which tends to work together to realize a certain function (Liu et al., 2018). We conducted relevant research to address the problem of group structure in the brain network (Guo et al., 2018). The elastic net and group lasso methods were introduced to construct a hypernetwork, the results of which showed that the elastic net method could better solve the group structure problem and achieve superior classification performance than the latter. The elastic net method could solve the group effecting problem (Zou and Trevor, 2005), because the l_2_ penalty leads to group effecting, i.e., it can tend to make highly correlated variables have similar regression coefficients with non-zero. However, it should be noted that this does not generally mean that highly correlated variables belong to the active set in the group (Sjöstrand et al., 2018).

There are multiple methods to create a hypernetwork. The scientific issues concerned in this article are mainly studied from the perspective of sparse representation methods. Based on the sparse representation method, different problems can be solved using different norm regularizations, such as the existing tree structure problem (Liu and Ye, 2010) and group structure problem (Ma et al., 2007) in the field of bioinformatics. In the current study, we mainly focused on whether the existence of group structures and different solutions of group structures will improve the diagnosis of brain diseases. Considering the existence of a potential group structure among the brain regions, we extended Guo et al.’s research (Guo et al., 2018) and further introduced the sparse group lasso (Friedman et al., 2010; Ogutu and Piepho, 2014; Matsui, 2018) method to solve the sparse regression model, improve the hypernetwork construction, and solve the group structure problem. The sparse group lasso method is a method of mixing lasso and group lasso, selecting both intergroup variables and variables in the group, which is a bi-level selection method. This method can effectively remove unimportant groups as well as unimportant individual variables within important groups (Friedman et al., 2010; Ogutu and Piepho, 2014). In other words, if there is a strong correlation between a specific brain region and several brain regions in a group, the specific brain region will not select the entire group, rather it will have several brain regions that are truly highly correlated. Thus, to prove the effectiveness of the proposed method, this article introduces the traditional lasso method, group lasso method, and sparse group lasso method to construct hypernetworks for related comparison.

Besides, in the previous study, only the clustering coefficient of a single node was involved as a feature extraction method, which is similar to the definition of the clustering coefficient in the conventional graph. However, multiple studies have shown a significant overlap between real network neighborhoods, wherein not only are neighbor nodes around individual vertices more likely to overlap but also that single sides have greater cohesiveness around individual edges (Goldberg and Roth, 2003; Latapy et al., 2008; Gallagher and Goldberg, 2013). Therefore, to more accurately clarify the mechanism of neuropsychiatric diseases and comprehensively evaluate disease performance, this study introduced the mutual clustering coefficients (clustering coefficients defined on a pair of nodes) that are widely used in hypernetworks as another feature extraction method (Goldberg and Roth, 2003; Estrada and Rodr Guez-Vel Zquez, 2006; Latapy et al., 2008; Gallagher and Goldberg, 2013). Subsequently, the non-parametric test method was used to select features with significant difference between the two types of clustering coefficient indicators, and the two sets of significant difference indicators were combined for multi-kernel learning, thereby improving the classification performance and providing more accurate and relevant imaging markers.

The main focus of this paper includes: (1) construction of the brain function hypernetwork by using the traditional lasso method, group lasso method, and sparse group lasso method; (2) extraction of features by using two types of hypernetwork clustering coefficients that express the brain functional network topology more fully, wherein features with differences were selected using non-parametric tests; and (3) use of multi-kernel SVM to classify significantly different features. The classification results revealed that the sparse group lasso method achieved the highest accuracy among these methods. In addition, based on these three methods, we analyzed the network topology and comparative analysis by using features with significant differences. Furthermore, we analyzed the influence of model parameters and classifier parameters on classification performance.

## Materials and Methods

### Method Framework

**FIGURE 1:**
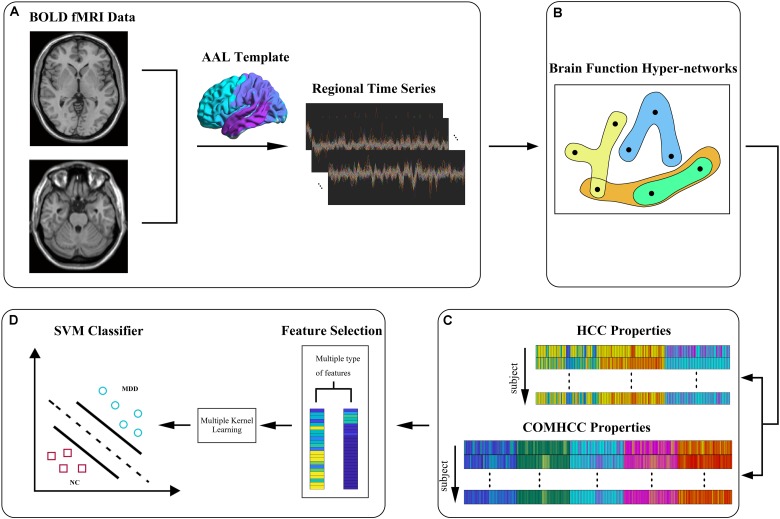
The flowchart of experimental process, including **(A)** data acquisition and preprocessing, **(B)** brain functional hypernetwork construction, **(C)** feature extraction based on two types of features, and **(D)** feature selection and classification.

The process framework and the construction and analysis of brain functional hypernetwork based on the sparse group lasso, traditional lasso, and group lasso methods mainly includes data collection and preprocessing, construction of the hypernetwork, feature extraction, feature selection, and classification. Figure 1 shows the entire flowchart. Specifically, this process consists of the following steps:

(1) Data collection and preprocessing.
(2) Construction of the hypernetwork: for each subject, we used a sparse linear regression model to create a hypernetwork, i.e., sparse learning (sparse group lasso method) was used to optimize the objective function and the selected region was represented by a linear combination of time series of other regions.

(3) Feature extraction and selection.

(3.1) We calculated the clustering coefficients defined on a single node using the definition in the traditional graph; in other words, we evaluated the proportion that node neighbors are neighbors of each other. The concept was applied to the hypernetwork in the same manner, and the local clustering coefficient of each node was obtained.
(3.2) Next, we calculated the clustering coefficients defined on pairs of nodes by using the definition of clustering coefficients commonly used in hypernetworks, i.e., we determined how many common edges are shared by a pair of nodes.
(3.3) Non-parametric tests were used to select brain regions from two different types of local clustering coefficients.

(4) Classification model construction.

(4.1) The corresponding classifier was constructed by classification features that combined the features with significant differences selected by two different types of local clustering coefficients.
(4.2) The cross-validation method was used to test the classifier and obtain the final classification result.

### Data Acquisition and Preprocessing

This study was performed according to the recommendations of the medical ethics committee of the Shanxi Province (reference number: 2012013). All participants signed a written agreement in light of the tenets of the Helsinki Declaration. A total of 66 participants were recruited, including 38 first-episode, drug-free patients with depression (15 male; mean age: 28.4 ± 9.68 years, range: 17–49 years) and 28 healthy subjects (13 male; mean age: 26.6 ± 9.4 years, range: 17–51 years). All subjects were right-handed. A resting-state fMRI scan was carried out for all subjects with a 3T magnetic resonance scanner (Siemens Trio 3-Tesla scanner, Siemens, Erlangen, Germany) (see Table 1 for details on subject’s information).

**TABLE 1:** Demographics and clinical characteristics of the study subjects.

	NC (*n* = 28)	MDD (*n* = 38)	*P*-value
Age (years)	17–51 (26.6 ± 9.35)	17–49 (28.4 ± 8.99)	0.41^a^
Gender (male/female)	13/15	15/23	0.55^b^
Handedness (R/L)	28/0	38/0	–
HAMD	NA	15-42 (22.8 ± 13.19)	–

*Data are presented as the range (mean ± SD). HAMD, Hamilton Depression Rating Scale; MDD, major depressive disorder. NA, not applicable; NC, normal controls. ^a^P-values was calculated by two-sample two-tailed t-test; ^b^P-value was computed by two-tailed Pearson’s χ^2^ test.*

Data acquisition was carried out at the First Hospital of Shanxi Medical University by radiologists familiar with MRI. During the scan, subjects were asked to relax and close their eyes, but remain awake. The scan parameters were set as follows: axial slices = 33, repetition time (TR) = 2000 ms, echo time (TE) = 30 ms, thickness/skip = 4/0 mm, field of view (FOV) = 192 × 192 mm, matrix = 64 × 64 mm, flip angle = 90°, and volumes = 248. Owing to the instability of the initial magnetic resonance signal and the adaptability of the subject to the environment, the time series of the first 10 functional images were discarded (see Supplementary Text S1 for detailed scan parameters).

The dataset was preprocessed using the SPM8 package^1^. Time slice correction and head movement correction were first performed. Then, two patient samples and two normal samples were discarded because of more than 3-mm head movement or 3 degrees of rotation during the correction process, which were not included in the 66 subjects’ dataset. The corrected image was obtained by 12-dimensional optimization affine transformation, which normalized to 3 mm × 3 mm × 3 mm voxels in the Montreal Neurological Institute (MNI) standard space. Linear dimensionality reduction and bandpass filtering (0.01–0.10 Hz) were finally performed to avoid low-frequency drift and high-frequency bio-noise.

### Construction of Hypernetwork

#### Hypergraph Graph

In neuroimaging, graph theory as a branch of mathematics has been widely used in brain network analysis, mainly to discretize the brain into different nodes and their interconnection edges (Sporns, 2012; Fornito et al., 2013). Most previous studies constructed network models using simple graphs, where the brain region was represented by nodes and the connections between two nodes was represented by an edge; this can only express pairwise correlation between brain regions. However, an increasing number of studies have proven that there is a higher-order relationship in the brain regions’ interactions. Therefore, to overcome this limitation, we introduced a hypernetwork, different from the single graph, where one hyperedge can connect more than two nodes. A simple graph was a special case of hypergraph, where every edge only connected two vertices. In brief, a hypergraph is an extension of a traditional graph model in which each hyperedge can be connected to any number of vertices. Compared with traditional graphs, hypergraphs focus more on relationships than nodes. This property of the hypergraph makes it easier to express multivariate relationships in complex networks. Hypergraphs have been applied in many fields such as social networks, food webs, reaction and metabolic networks, neural networks, protein-protein interaction networks, collaboration network, and other application fields because of the advantages it offers of exploring complex variable relationships (Mäkinen, 1990). In real network, a large number of data objects are not independent and have complex and diverse associations among them. Several studies have found that multivariate relationships can more naturally express the hidden internal connections and patterns in information (Estrada and Rodr Guez-Vel Zquez, 2006). Supplementary Figure S1 shows an example of a hypergraph.

From the point of view of a mathematical expression, the hypergraph can be represented by *H = (**V,E**)* (Kaufmann et al., 2016), in which ***V*** = {*v*} represents the set of vertices, ***E*** = {*e*} represents the hyperedge set, and the hyperedge *e* ∈ *E* is a subset of V. The hypergraph can be represented by a | ***V***| × | ***E***| incidence matrix H and is defined as follows:

(1) $$H\,\left(v,e\right)=\left\{ \begin{array}{c} 1,i\,f\,v\in e\\ 0,i\,f\,v\notin e \end{array}\right.$$

where *H*(*v*,*e*) represents the corresponding element in the incidence matrix, *v* ∈ *V* represents the node, *e* ∈ *E* represents the hyperedge, row element of the incidence matrix refers to the node, and the column element refers to the hyperedge. If the node *v* belongs to the hyperedge *e*, then *H*(*v*,*e*) = 1, and conversely, if the node v does not belong to hyperedge e, then *H*(*v*,*e*) = 0.

For vertex *v* ∈ *V*, its node degree based on H is defined as:

(2) $$d\,\left(v\right)=\sum_{e\in E}H\,\left(v,e\right)$$

Similarly, the edge degree of the hyperedge *e* ∈ *E* is expressed as:

(3) $$\delta \,\left(e\right)=\sum_{v\in V}H\,\left(v,e\right)$$

*D*_*v*_ and *D*_*e*_ represent the diagonal matrices of node degrees *d(v)* and hyperedge degrees δ*(e)*, respectively.

#### Sparse Linear Regression Model

In this study, the brain region was divided into 90 anatomical regions of interest (ROIs) according to the anatomical automatic labeling (AAL) (Tzourio-Mazoyer et al., 2002) template (45 ROIs per hemisphere), with each ROI representing a node in the functional brain network (except for the cerebellum). The average time series for each region was obtained by regression of mean cerebrospinal fluid (CSF) and white matter signals as well as six parameters from motion correction. The functional hypernetwork was constructed using linear regression methods (Jie et al., 2016) based on rs-fMRI time series. By using a sparse linear regression model, one region could be represented as a linear combination of other regions, which exhibited an interaction of a particular region with other regions, while forcing a meaningless or false interaction to be zero.

The average time series of *m*-th ROI for *n*-th subject, $x_{m}^{n}=A_{m}^{n}\alpha_{m}^{n}+\tau_{m}^{n}$, can be viewed as a response vector, which can be estimated as a linear combination of time series of other ROIs. The sparse linear regression model is specifically expressed as follows:

(4) $$x_{m}^{n}=A_{m}^{n}\,\alpha_{m}^{n}+\tau_{m}^{n}$$

where $x_{m}^{n}=[x_{m}^{n}(1);x_{m}^{n}(2);\ldots ;x_{m}^{n}(T)]$ refers to the average time series of the *m*-th ROI for *n*-th subjects, with T being the number of time points in the time series; $A_{m}^{n}=[x_{1}^{n},\ldots ,x_{m-1}^{n},0,x_{m+1}^{n}\ldots ,x_{M}^{n}]$ denotes the data matrix of the *m*-th ROI (all the average time series except for the *m*-th brain region, and the average time series of the *m*-th ROI being set to 0); $\alpha_{m}^{n}=[\alpha_{1}^{n},\ldots ,\alpha_{m-1}^{n},0,\alpha_{m+1}^{n}\ldots ,\alpha_{M}^{n}]$ denotes the coefficient vector that quantifies the degree of influence from the other ROI to the *m*-th ROI; and $\tau_{m}^{n}$ denotes a noise term, being Gaussian. The ROIs corresponding to the non-zero element in $\alpha_{m}^{n}$ are the ROIs interacting with the particular ROI; by contrast, the corresponding ROI of the zero element is conditionally independent with the *m*-th ROI.

#### Construction of Hypernetwork Based on Lasso Method

In existing literature, the brain function hypernetwork is constructed by using the lasso method to solve the sparse linear regression model (Jie et al., 2016), and its optimization objective function is as follows:

(5) $$m\,i\,n_{\alpha_{m}}\,{||x_{m}^{n}-A_{m}^{n}\,\alpha_{m}^{n}||}_{2}+\lambda \,{||\alpha_{m}^{n}||}_{1}$$

This is a well-known NP problem, which is usually estimated by solving the l_1_ norm problem, and $x_{m}^{n}$, $A_{m}^{n}$, and $\alpha_{m}^{n}$ have the same meaning as in equation (4). ‖.‖_2_ refers to the l_2_ norm, ‖.‖_1_ refers to the l1 norm, and λ > 0 refers to a regularization parameter to control the sparsity of the connection matrix. It is worth noting that different λ values correspond to different sparsity. If the λ value is larger, the connection network is sparser; that is, there are more zeros in the $\alpha_{m}^{n}$. On the contrary, the connection network is denser when the λ value is smaller; in other words, there are more non-zeros in the $\alpha_{m}^{n}$. Thus, λ requires a range. However, different experimental data will have different λ ranges. In our experiment, the lasso, gLasso, and sgLasso methods in the SLEP package (Liu et al., 2013) were used to solve the optimization problem. In this software package, in order to avoid the difficulty of regularization parameter selection, parameter control is added to λ value. The λ should be specified as a ratio whose value lies in the interval (Zeng et al., 2012), but the actual regularization value is λ = λ × *λ_*max*_*, where *λ_*max*_* is computed such that 0 resides in the subgradient (set) at 0; that is, the solution is all zeros. *λ_*max*_* is different under different methods, which is dependent on the regularization used. Based on the average time series, the lasso method is executed to indicate the interaction between this node and its neighbors. Specifically, for a specific brain region, based on the time series, by fixing λ value, and a weight vector $\alpha_{m}^{n}$ will be generated, the brain region corresponding to non-zero elements in $\alpha_{m}^{n}$ and this brain region form a hyperedge. Further, in order to reflect the multi-level interactive information of the brain regions, for a centroid ROI, the λ value (0.1, 0.9) is changed to generate a set of hyperedges. Then each brain region is regarded respectively as a specific brain region to calculate their corresponding hyperedges. Finally, the hyperedges corresponding to all brain regions constitute a hypernetwork, which is a matrix of 90 ^∗^ 810 for each subject.

#### Construction of Hypernetworks Based on the Group Lasso Method

Although the lasso method has been successfully applied in many fields, it has limitations. The lasso often randomly chooses only one variable from a group of several highly correlated variables (Zou and Trevor, 2005). To elaborate, when choosing a group of more relevant brain regions, the particular brain region tends to choose one brain region with a group structure and regardless of which one, which results in some correlated brain regions not being selected, eventually leading to a poor ability to interpret group structure information. The ideal hyperedge construction method should be able to select the interacting brain regions as accurately as possible. To solve this problem, we considered the group structure problem among brain regions in our previous research, and introduced the group lasso method to improve construction of the hypernetwork.

The group lasso method is a generalization of the lasso method (represented by gLasso) and is based on a linear regression model. This method efficiently carries out variable selection on the basis of a predefined set of variables (Meier et al., 2008) to solve the limitation that only selects a single variable based on the lasso method. Because the gLasso method selects variables based on group level, a clustering method was needed to distinguish the strongly related brains into a group before using the gLasso method to create a hypernetwork; the method was then used to construct the hyperedge. In other words, when the hypernetwork is constructed, we must first cluster according to the average time series of ROIs to obtain the grouping relationship of 90 brain regions. Here, we used the k-medoids algorithm (Park and Jun, 2009), where the pairwise similarity between brain regions was first computed: the larger the value, the more similar the two samples are. When clustering, all brain regions were divided into *k* groups, where each group meant a class of objects and the relationship between objects and groups had to satisfy the following conditions: (1) each group implied at least one object and (2) each object belonged to a group. Moreover, the k-means++ (Arthur and Vassilvitskii, 2007) was adopted to select the *k* initial cluster centers to ensure the stability of the cluster. A point was randomly selected as the first initial cluster center, and then the replacement center was randomly selected from the remaining data points with a probability that was proportional to the distance of the data point from the nearest cluster center point. Clustering was repeated 10 times to select the best clustering effect as the final result. It is noteworthy that the setting of k in clustering affects network topology and classification performance. In this study, we observed that the gLasso method achieved the highest classification accuracy when k was set to 48 (detailed analysis mentioned in Methodology section). Then, the gLasso method was used to construct the hypernetwork by solving the sparse linear regression model. The following is the optimization objective function:

(6) $$m\,i\,n_{\alpha_{m}}\,{||x_{m}^{n}-A_{m}^{n}\,\alpha_{m}^{n}||}_{2}+\beta \,\sum_{i=1}^{k}{||\alpha_{m}^{n}\,G_{i}||}_{2,1}$$

where β is l_2, 1_-norm regularization parameter, which is a critical value of l_1_-norm and l_2_-norm penalty that can be used to make variable selections at the group level (Yuan and Lin, 2006; Friedman et al., 2010). Different β values correspond to different sparsity. If the β value is larger, the model is sparser and fewer groups are selected. To elaborate, in case of a group of brain regions where their pairwise correlations are comparatively high, the gLasso model tends to regard all brain regions of the group as a whole to decide whether it is important for the problem. $\alpha_{m}^{n}$ s classified into k non-overlapping groups by clustering, and $\alpha_{m}^{n}\,G_{i}$ indicates the *i*-th group. In the same way, the hyperedges were constructed based on the ROIs corresponding to the non-zero elements in $\alpha_{m}^{n}$; that is, hyperedges denote the centroid ROI and fewer other ROIs, and the node was represented by ROI. A hyperedge was produced in a selected ROI, and a group of hyperedges were generated by varying the lambda value from 0.1 to 0.9 in increments of 0.1 for a particular ROI. Accordingly, the hypernetwork was constructed based on the gLasso method by considering every ROI as a centroid ROI. Finally, a 90^∗^810 matrix is generated; that is, a hypernetwork is constructed for a subject.

#### Construction of Hypernetwork Based on the Sparse Group Lasso Method

From the above research mentioned, gLasso considers the entire group as a whole and determines whether it is essential to the problem. Although the gLasso method lists a set of sparse groups, if a group is included in the model, all coefficients in that group will be non-zero. Sometimes, we preferred to include both groupwise sparsity and intragroup sparsity. For example, if an ROI is used as the predictor, some particularly “important” brain regions in multiple brain region interactions should be identified as accurately as possible. However, this method does not generate sparsity within a group. That is, several brain regions with a group structure in the brain functional hypernetwork have a high correlation with the selected brain regions, but the gLasso method considers that all brain regions in the group are non-zero; in other words, all brain regions were believed to have a high correlation with selected brain regions in the gLasso method. Thus, the hypernetwork based on the gLasso method is rather loose, there are likely many fake connections, or some useful connections are lost.

Therefore, the sparse group lasso (represented by sgLasso) (Friedman et al., 2010) method was introduced to create a hypernetwork. This method is still based on a linear regression model. The variable in this method was selected not only at the group level but also at a single variable level; that is, variables within groups and groups can be freely chosen. In a functional brain hypernetwork, if a particular ROI is correlated highly with one or several brain regions with a group structure, the method does not select all ROIs in the group, rather only one or more brain regions of the group associated with a selected ROI. Indeed, if the group is highly correlated with a centroid ROI, the entire group will be selected, such that some fake or false connections can be filtered out and some useful connections retained.

Similar to the gLasso method, clustering was adopted before creating the hyperedge, and then the sgLasso method was used to construct the hyperedge by solving the sparse linear regression model. The method is represented by the optimizationobjective function:

(7) $$\begin{array}{c} m\,i\,n\\ \alpha_{m} \end{array}\,{∥x_{m}^{n}-A_{m}^{n}\,\alpha_{m}^{n}∥}_{2}+\lambda_{1}\,{∥\alpha_{m}^{n}∥}_{1}+\lambda_{2}\,\sum_{i=1}^{k}{∥\alpha_{m}^{n}\,G_{i}∥}_{2}$$

$\alpha_{m}^{n}$ is divided into k non-overlapping tree groups ($\alpha_{m}^{n}$*G*_1_,$\alpha_{m}^{n}$*G*_2_,…,$\alpha_{m}^{n}$*G*_*k*_) by clustering, and *G*_*i*_ is a node with tree structure. λ_1_ and λ_2_ are regression parameters, with λ_1_ being used to adjust the sparsity of intra-groups to control the number of non-zero coefficients in non-zero groups, and λ_2_ being used to adjust group-level sparsity (Yuan and Lin, 2006; Friedman et al., 2010) to control the number of groups with non-zero coefficients. This model is a combination of traditional lasso and gLasso. The gLasso estimate is obtained when λ_1_ = 0, and the lasso estimate is acquired when λ_2_ = 0. It should be noted that the model looks somewhat similar to the elastic net model, but it is different because the l_2_ penalty is not differentiated at 0, so some groups are completely zeroed. However, in each non-zero group, it performs an elastic net fit (Simon et al., 2013). Like the gLasso method, a hypernetwork was constructed for each subject, where the ROI was regarded as the node, and the hyperedge comprised the *m*-th ROI and the ROIs corresponding to the zero elements in $\alpha_{m}^{n}$. For each ROI, a set of hyperedges were produced by fixing the λ_2_ value and varying the λ_1_ value from 0.1 to 0.9 in increments of 0.1. Finally, a hypernetwork is a 90*810 matrix. In this experiment, the sgLasso method achieved the highest accuracy (87.12%) of all three models, when λ_2_ was equal to 0.4. (see the Methodology Section about relative analysis).

### Feature Extraction and Selection

After the functional connection hypernetwork being created, it was necessary to select a representative feature set that could identify the target. This required feature definition and selection of the property value of each vertex in the hypernetwork as the feature. In the hypernetwork analysis of brain function, there are many indicators that can reflect the characteristics of nodes and the whole network. However, in the field of medical imaging, most studies use the clustering coefficient as a local attribute index to improve diagnostic performance and identify biomarkers associated with disease pathology. In our previous study, the clustering coefficient defined on a single node was only involved as a feature extraction method. However, according to several studies, there is a significant overlap between neighborhoods in the real network, meaning in addition to the neighboring nodes between individual vertices being more likely to overlap, the individual edges also show greater cohesiveness (Goldberg and Roth, 2003; Latapy et al., 2008; Gallagher and Goldberg, 2013). Therefore, to accurately and comprehensively evaluate disease performance, this study introduced the mutual clustering coefficient defined on pairs of nodes that have been widely applied in the hypernetwork as another feature extraction method. The specific definition is as follows:

#### Feature Extraction Based on Clustering Coefficients Defined by Single Nodes

After the hypernetwork model was completed by using the above three methods, feature extraction was required for three hypernetworks. From different views, we introduced the clustering coefficients (HCC^1^, HCC^2^, HCC^3^) of three different definitions in the hypergraph to describe the local aggregation of the hypernetwork (Gallagher and Goldberg, 2013). The clustering coefficients were defined on a single vertex, which was the same as that of the traditional graph; specifically, we determined what proportion of a node’s neighbors are neighbors of each other. The first type of clustering coefficient, HCC^1^ (v), captures the number of adjacent nodes that have connections not facilitated by node *v*. The second type, HCC^2^ (v), emphasizes the number of adjacent nodes that have connections facilitated by node *v*. The third type, HCC^3^ (v), denotes the amount of overlap amongst adjacent hyperedges of node *v*. The formula is as follows:

(8) $$H\,C\,C^{1}\,\left(v\right)=\frac{2\,\sum_{u,t\in N\,\left(v\right)}I\,\left(u,t,\neg \,v\right)}{\left|N\,\left(v\right)\right|\,\left(\left|N\,\left(v\right)-1\right|\right)}$$

(9) $$H\,C\,C^{2}\,\left(v\right)=\frac{2\,\sum_{u,t\in N\,\left(v\right)}I^{\prime }\,\left(u,t,v\right)}{\left|N\,\left(v\right)\right|\,\left(\left|N\,\left(v\right)\right|-1\right)}$$

(10) $$H\,C\,C^{3}\,\left(v\right)=\frac{2\,\sum_{e\in S\,\left(v\right)}\left(\left|e\right|-1\right)-\left|N\,\left(v\right)\right|}{\left|N\,\left(v\right)\right|\,\left(\left|S\,\left(v\right)\right|-1\right)}$$

where *u, t*, and *v* represent nodes; *N*(*v*) = {*u* ∈ ***V*** : ∃*e* ∈ **E**, *u*, *v* ∈ *e*}, where ***V*** refers to the set of nodes, ***E*** refers to the set of hyperedges, *e* refers to hyperedge, and *N(v)* refers to a set of other nodes included in the hyperedge containing node *v*; when *∃*e*_*i*_ ∈ *E** and *u*,*t* ∈ *e*_i_, but *v*∉*e*_i_, then *I*(*u*, *t*, ¬*v*) = 1, if not, then *I*(*u*, *t*, ¬*v*) = 0; *S*(*v*) = {*e*_i_ ∈ *E* : *v* ∈ *e*_i_}, in which v represents a node, *e*_i_ represents a hyperedge, and *S*(*v*) represents the set of hyperedges containing node *v*.

These three clustering coefficients reflect the local clustering properties based on a single vertex of the hypernetwork from different angles. For each clustering coefficient definition, we separately extracted features from the connectivity hypernetwork. Multiple linear regression analyses were performed to measure the influence of confounding variables such as age, sex, and educational status on each network property. As the three clustering coefficients refer to local attributes of hypernetwork, for the sake of simplicity, the average clustering coefficient (average HCC^1^, average HCC^2^, and average HCC^3^) of each subject (averaged for 90 brain regions) was calculated as an independent variable for the multivariate linear regression, where insignificant correlation was expressed between network indicators and confounding variables (see Supplementary Text S2 for details).

#### Feature Extraction Based on Clustering Coefficients Defined by Pairs of Nodes

Multiple studies have demonstrated that real networks can be represented by small world network, and there is a significant overlap between their neighbors. In this real network, not only are neighbor nodes between individual vertices more likely to overlap, but they also show greater neighborhood cohesiveness around individual edges (Goldberg and Roth, 2003; Latapy et al., 2008; Gallagher and Goldberg, 2013). Therefore, clustering coefficients between pairs of nodes are defined by the extension of traditional clustering coefficients; that is, the number of how many common edges a pair of nodes share. This method has been widely applied in the fields of hypernetworks (Gallagher and Goldberg, 2013). In the hypergraph analysis, several methods have been proposed to calculate the clustering coefficients based on pairs of nodes (Goldberg and Roth, 2003; Estrada and Rodr Guez-Vel Zquez, 2006; Latapy et al., 2008; Gallagher and Goldberg, 2013). In our study, we introduced several clustering coefficients based on pairs of nodes that have been widely used in hypergraph research to comprehensively assess diagnostic performance and better identify biomarkers associated with disease pathology. Table 2 presents the definitions and calculation formulae for these characteristics.

**TABLE 2:** Definitions and calculation formulae of clustering coefficients defined on a pair of nodes.

Properties	Definitions	Formulas
*COMHCC*^1^	*COMHCC*^1^(*u*,*v*) emphasizes the overlap between neighborhoods of nodes: when *u* and *v* have no adjacent edges in common, then COMHCC^1^ (*u, v*) = 0. When they have the same adjacent edges, then COMHCC^1^(*u, v*) = 1. When their adjacent edges partially overlap, then the value is in between 0 and 1 (Latapy et al., 2008).	$C\,O\,M\,H\,C\,C^{1}\,\left(u,v\right)=\frac{\left|S\,\left(u\right)\,\cap S\,\left(v\right)\right|}{\left|S\,\left(u\right)\,\cup S\,\left(v\right)\right|}$
*COMHCC*^2^	*COMHCC*^2^(*u*,*v*) emphasizes the fact that neighborhoods (both small or large ones) may overlap very significantly: it is 1 only when the two neighborhoods are the same and it often decreases rapidly if the degree of one of the involved nodes increases. It captures the fact that nodes with similar degrees have high neighborhood overlaps (Latapy et al., 2008).	$C\,O\,M\,H\,C\,C^{2}\,\left(u,v\right)=\frac{\left|S\,\left(u\right)\,\cap S\,\left(v\right)\right|}{\max\,\left\{\left|S\,\left(u\right)\right|,\left|S\,\left(v\right)\right|\right\}}$
*COMHCC*^3^	*COMHCC*^3^(*u*,*v*) captures the fact that small neighborhoods may intersect significantly large ones; it is equal to 1 whenever one of the neighborhoods is included in the other (Latapy et al., 2008).	$C\,O\,M\,H\,C\,C^{3}\,\left(u,v\right)=\frac{\left|S\,\left(u\right)\,\cap S\,\left(v\right)\right|}{\min\,\left\{\left|S\,\left(u\right)\right|,\left|S\,\left(v\right)\right|\right\}}$
*COMHCC*^4^	*COMHCC*^4^(*u*,*v*) is an intermediate between the meet/max and the meet/min standards (Gallagher and Goldberg, 2013).	$C\,O\,M\,H\,C\,C^{4}\,\left(u,v\right)=\frac{\left|S\,\left(u\right)\,\cap S\,\left(v\right)\right|}{\sqrt{\left|S\,\left(u\right)\right|\,\left|S\,\left(v\right)\right|}}$
*COMHCC*^5^	*COMHCC*^5^(*u*,*v*) can be interpreted as a *p-*value; the probability of obtaining a number of mutual neighbors between vertices *v* and *w* at or above the observed number by chance (Gallagher and Goldberg, 2013)	$C\,O\,M\,H\,C\,C^{5}\,\left(u,v\right)=-l\,o\,g\,\sum_{i=\left|S\,\left(u\right)\,\cap S\,\left(v\right)\right|}^{\min\,\left\{\left|S\,\left(u\right)\right|,\left|S\,\left(v\right)\right|\right\}}\frac{\left(\begin{array}{c} \left|S\,\left(u\right)\right|\\ i \end{array}\right)\,\left(\begin{array}{c} T\,o\,t\,a\,l-\left|S\,\left(u\right)\right|\\ \left|S\,\left(v\right)\right|-i \end{array}\right)}{\left(\begin{array}{c} T\,o\,t\,a\,l\\ \left|S\,\left(v\right)\right| \end{array}\right)}$

*u and v represent nodes, S(v) = {e_i_ ∈ E:v ∈ e_i_}, where v represents a node, e_i_ represents a hyperedge, S(v) represents the set of hyperedges containing node v and Total represents total number of hyperedges.*

After calculating the clustering coefficients of the pairs of nodes, the clustering coefficients of the single node are obtained by averaging the clustering coefficients of the node and all its neighboring nodes (Latapy et al., 2008).

(11) $$\mathrm{COMHCC}\,\left(v\right)=\frac{\sum_{u\in N\,\left(v\right)}C\,O\,M\,H\,C\,C\,\left(u,v\right)}{\left|N\,\left(v\right)\right|}$$

COMHCC(*u,v*) refers to the clustering coefficient between pairs of nodes by the above method. *N*(*v*) = {*u* ∈ *V* : ∃*e* ∈ *E*, *u*, *v* ∈ *e*}, where *V* represents the set of nodes, *E* represents the set of edges, *e* represents a hyperedge, and *N*(*v*) represents the collection of other nodes contained in the hyperedge included in the node *v*.

The clustering coefficients defined on pairs of nodes reflected the neighborhood cohesiveness around individual edges from different angles; next, the clustering coefficient of each node was calculated to more fully express the local clustering property of the hypernetwork. According to the clustering coefficient definition, we separately extracted the features from the connectivity hypernetwork. Similarly, multiple linear regression analyses were carried out to evaluate the effects of confounding variables (age, sex, and educational status) for each network index. To simply calculate, the average clustering coefficients were computed (mean COMHCC^1^, mean COMHCC^2^, mean COMHCC^3^, mean COMHCC^4^, and mean COMHCC^5^) for each subject (averaged for 90 brain regions) as independent variables for further multivariate linear regression. The results showed that significant correlation had not been found between the clustering coefficients based on two nodes and confounding variables (see Supplementary Text S3 for relative results).

#### Feature Extraction

Features extracted from a hypernetwork may contain some irrelevant or redundant information. Therefore, to select key features for classification, the most discriminative features were selected according to different statistical analysis. For patients with major depressive disorder (MDD) patients and normal control (NC) subjects, the Kolmogorov–Smirnov non-parametric test was performed (Fasano and Franceschini, 1987) for 270 and 450 node attributes generated by clustering coefficients extracted by two different types, which was further corrected using the Benjamini and Hochberg false-discovery rate (FDR) method (*q* = 0.05) (Benjamini and Hochberg, 1995). After the Kolmogorov–Smirnov (KS) non-parametric permutation test, the local properties with significant difference were fused by multi-kernel learning as a classification feature to construct the classification model.

### Classification and Feature Validation

The classification model was constructed by using the local attributes of the hypernetworks with significant differences, which were regarded as input features in the classification model construction process. In this paper, we combined the selected classification input features and used the support vector machine (SVM) classification algorithm to construct the classifier model and classify the experimental data. Subsequently, we used cross-validation to evaluate the classification performance.

The MDD classification was performed by providing complementary information each other by two different types of clustering coefficients. Technically, integrating multi-features can improve the classification performance (Huang et al., 2019). As mentioned in Zhang et al. (2011), kernel based feature combinations using multi-kernel learning provide more flexible feature fusion by estimating different weights of features from different modalities, which can provide better methods from different types of clustering coefficients (De Bie et al., 2007). Typically, kernel integration uses a linear combination of multiple kernels:

(12) $$k\,\left(x,y\right)=\sum_{i=1}^{M}a_{i}\,k_{i}\,\left(x,y\right)\;s.t\,\sum_{i=1}^{M}a_{i}=1$$

where *k*_i_(*x*,*y*) represents the centered kernel function between subjects *x* and *y* in the clustering coefficient of the i-th type. M denotes the number of kernel matrices we built (*M* = 2), and *a*_i_ denotes a non-negative weight parameter. Here, multi-kernel learning was adopted to effectively fuse features from two different types of clustering coefficients by combining multiple kernels into one mixed kernel. Then, traditional a SVM classifier was used to classify the mixed kernel based on the libsvm package^2^.

The leave-one-out cross-validation (LOO-CV) method was used to evaluate classification performance. For example, if there were N samples, each sample was used as a test set, and the remaining N−1 samples were used as the training set. The classification set was finalized by establishing different models (N), and the average of the classification accuracy of the N models was considered the classification result. It should be noted that classification features need to be normalized before obtaining the classification model. In the gLasso or sgLasso-based methods, because the initial random selection of the seed points during clustering might affect the final classification result, we performed 50 experiments to select the arithmetic mean as the final classification result.

## Results

### Functional Network Topology Comparison Among Three Methods

**FIGURE 2:**
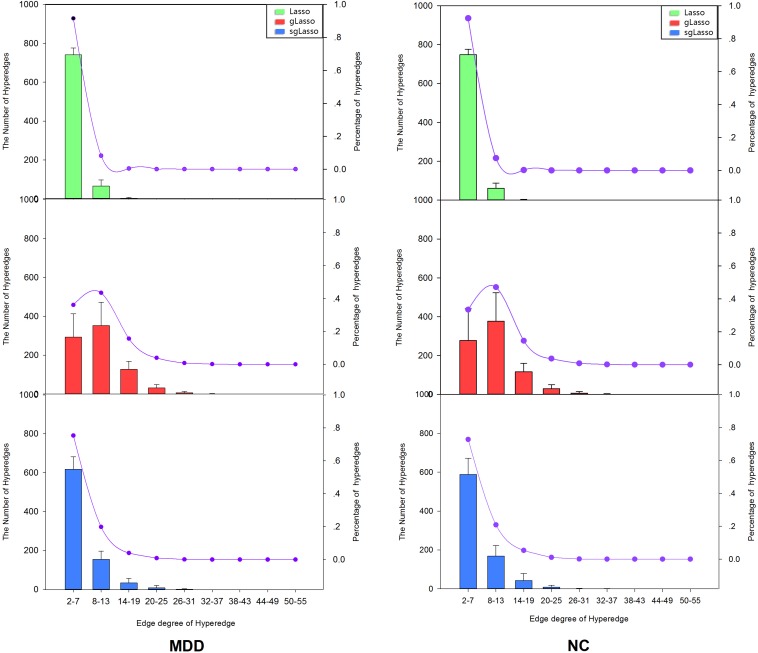
The distribution of the edge degree about hypernetwork obtained by three methods.

To infer whether there were significant differences among the hypernetworks constructed based on the traditional lasso, gLasso, and sgLasso methods, we carried out the following analysis:

The subjects were selected from both the normal and MDD groups, in which hyperedges were analyzed. The degree of edges of the hyperedges was calculated based on the three methods, whose distributions are shown in Figure 2. The results revealed that the ratio of the hyperedge degree distribution among three methods was different, both in the MDD and normal control groups. The hyperedges constructed by the lasso method are mostly distributed in the range of 2–7 (NC group: 2–7[92%], 8–13[7%]; MDD group: 2–7[91%], 8–13[8%]), where the distribution is relatively narrow. On the contrary, the range of hyperedge degree distribution in the gLasso method is mainly 2–19 (NC group: 2–7[33%], 8–13[47%], 14–19[15%]; MDD group: 2–7[36%], 8–13[43%], 14–19[16%]). The results showed that some hyperedges usually contain more nodes and their network is relatively loose. But the edge degree in the sgLasso method mostly lay in the range 2–13 (NC group: 2–7[72%], 8–13[21%]; MDD group: 2–7[75%], 8–13[19%]), which showed the distribution is relatively temperate.

**FIGURE 3:**
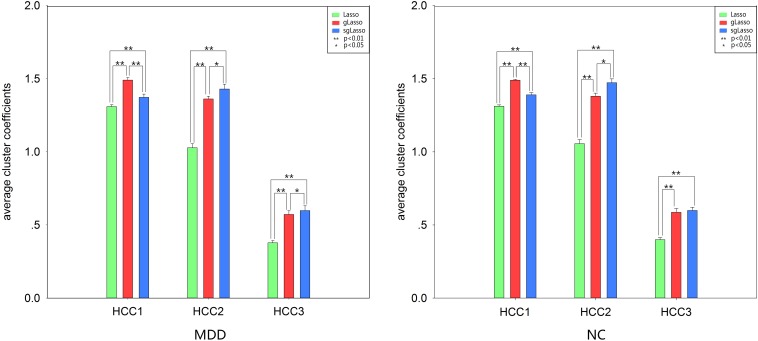
Comparison among three hypernetworks based on three kinds of average clustering coefficient for HCC properties. Error bars indicate standard deviation. Asterisks represent a significant difference; * *p* < 0.05, ** *p* < 0.01. Green histograms denote lasso method; Red histograms denote gLasso method; Blue histograms denote sgLasso method. NC, normal control; MDD, major depressive disorder.

**FIGURE 4:**
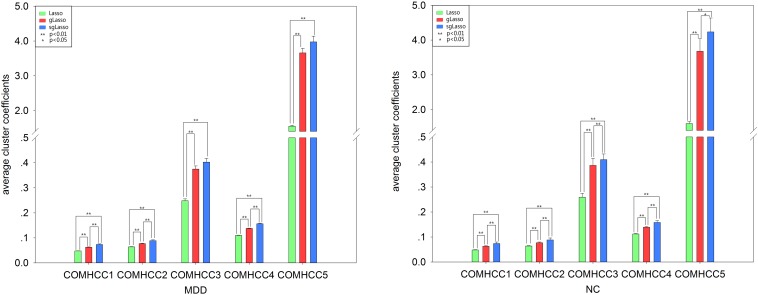
Comparison among three hypernetworks about three kinds of average clustering coefficient for COMHCC properties. Error bars indicate standard deviation. Asterisks represent a significant difference; * *p* < 0.05, ** *p* < 0.01. Green histograms denote lasso method; Red histograms denote gLasso method; Blue histograms denote sgLasso method. NC, normal control; MDD, major depressive disorder.

The average clustering coefficient (HCC^1^-HCC^3^ and COMHCC^1^-COMHCC^5^; averaged for 90 brain regions) was computed for each subject, and non-parametric permutation tests were implemented to compare hypernetwork differences among the three methods by using the average clustering coefficient (HCC^1^-HCC^3^, COMHCC^1^-COMHCC^5^) in the MDD and NC groups separately, which was further corrected by the FDR method. Figures 3, 4 show the average clustering coefficients of the three hypernetworks in the two groups’ feature extraction methods, respectively, which showed that there were differences in the three functional hypernetworks.

**FIGURE 5:**
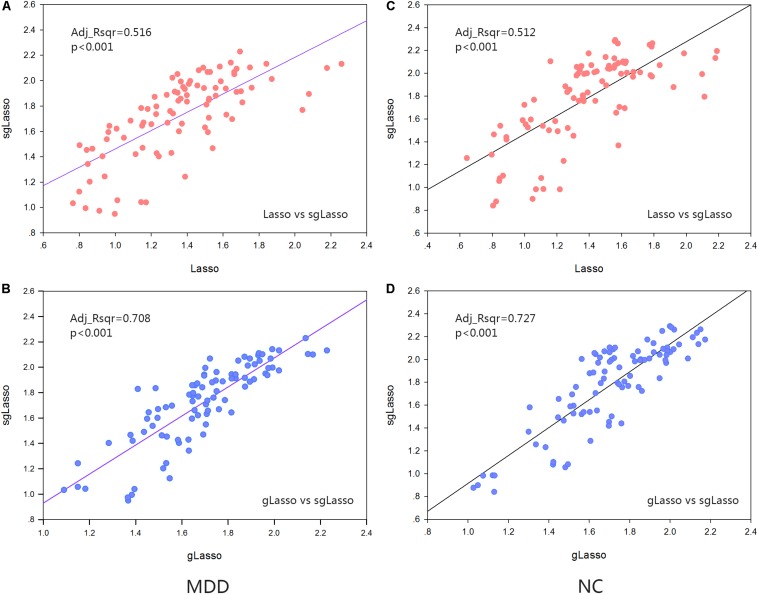
Regression analysis about the standardized metric values about the HCC metrics. **(A)** Regression analysis from that obtained by sparse group lasso and lasso method in the MDD group. **(B)** Regression analysis from that obtained by sparse group lasso and group lasso method in the MDD group. **(C)** Regression analysis from that obtained by sparse group lasso and lasso method in the NC group. **(D)** Regression analysis from that obtained by sparse group lasso and group lasso method in the NC group.

**FIGURE 6:**
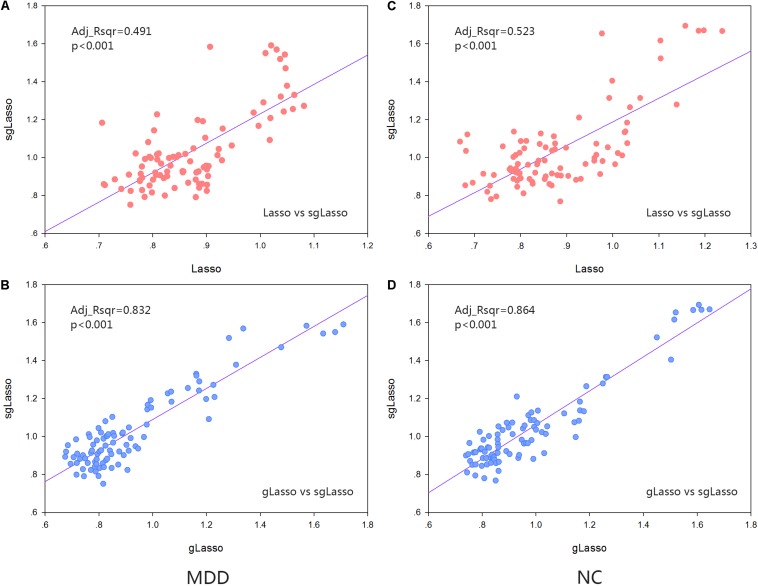
Regression analysis about the standardized metric values about the COMHCC metrics. **(A)** Regression analysis from that obtained by sparse group lasso and lasso method in the MDD group. **(B)** Regression analysis from that obtained by sparse group lasso and group lasso method in the MDD group. **(C)** Regression analysis from that obtained by sparse group lasso and lasso method in the NC group. **(D)** Regression analysis from that obtained by sparse group lasso and group lasso method in the NC group.

For each brain region, the mean cluster coefficient of each group of (NC and MDD) subjects under the hyper-network constructed by the three methods was calculated for each type of cluster coefficient, and the obtained data was normalized. Regression analysis was performed by the sgLasso and other two methods to verify the association of the network indicators obtained by all three methods. The results indicated that the sgLasso method had the strongest correlation with the traditional gLasso method and a weak association with the traditional lasso method (Figures 5, 6).

### Differential Brain Region

After hypernetwork construction and features extraction based on the traditional lasso, gLasso, and sgLasso methods, a non-parametric permutation test was performed using the extracted features to evaluate differences between the MDD and NC groups, and the result was corrected using the FDR method.

**FIGURE 7:**
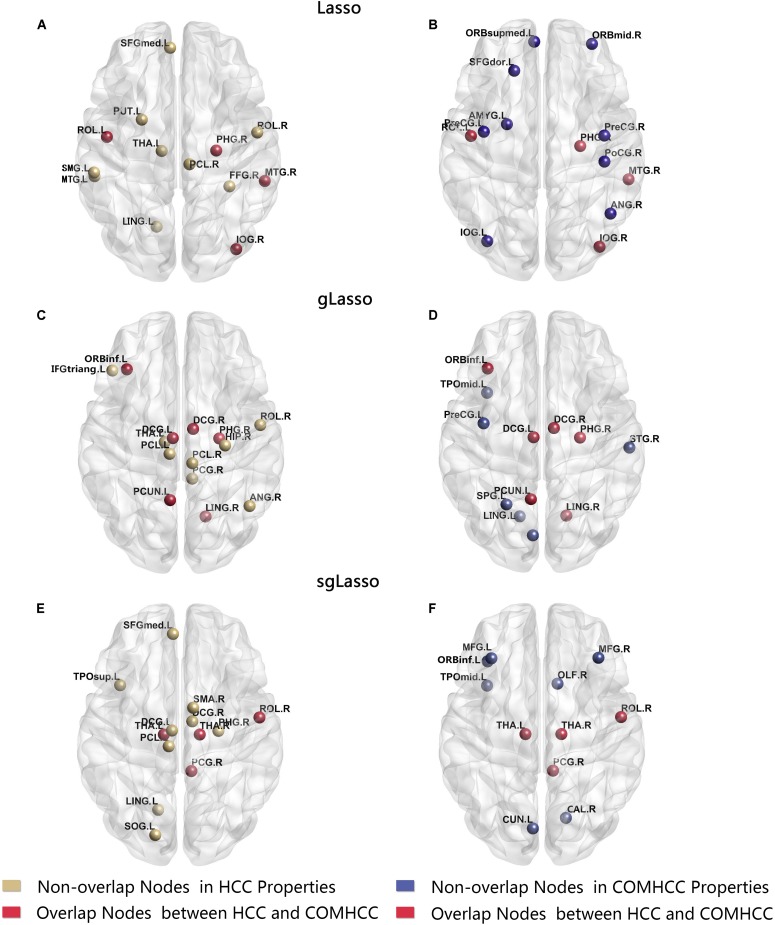
Abnormal brain regions were mapped onto the cortical surfaces using BrainNet viewer software. **(A)** Abnormal brain regions calculated by HCC properties in lasso method. **(B)** Abnormal brain regions calculated by COMHCC properties in lasso method. **(C)** Abnormal brain regions calculated by HCC properties in gLasso method. **(D)** Abnormal brain regions calculated by COMHCC properties in gLasso method. **(E)** Abnormal brain regions calculated by HCC properties in sgLasso method. **(F)** Abnormal brain regions calculated by COMHCC properties in sgLasso method.

**FIGURE 8:**
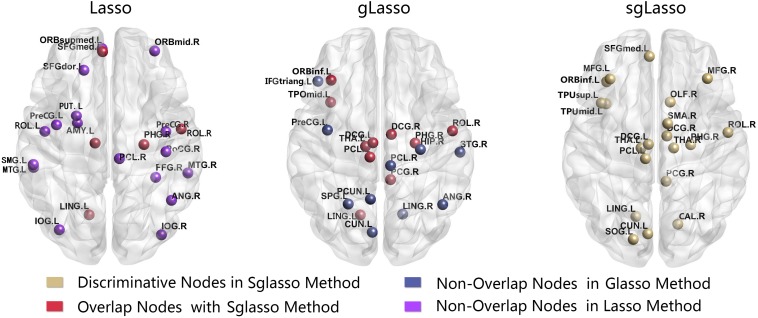
All abnormal brain regions were mapped onto the cortical surfaces using BrainNet viewer software.

Table 3 lists the brain regions computed by two different types of clustering coefficients based on these three methods that showed significant differences. There were fewer overlapping regions obtained by two sets of clustering coefficients in every method. The Lasso method mainly focuses on the left central sulcus, partial limbic lobe (right parahippocampal gyrus), partial occipital lobe (right inferior occipital gyrus), and partial temporal lobe (right middle temporal gyrus). The gLasso method mainly concentrates on the partial frontal lobe (left inferior frontal gyrus); partial limbic lobe (left median cingulate and paracingulate gyri, right median cingulate and paracingulate gyri, right parahippocampal gyrus, left precuneus); and partial occipital lobe (right lingual gyrus); while the sgLasso method mainly focuses on the partial parietal lobe (right central sulcus), part of the limbic lobe (right posterior cingulate gyrus), and bilateral thalamus (Figure 7). Meanwhile, all different brain regions obtained by the two clustering coefficients were compared, in which the sgLasso and gLasso methods showed greater overlap, including in the partial frontal lobe (left inferior frontal gyrus) and partial parietal lobe (right central sulcus); partial limbic lobe (left median cingulate and paracingulate gyri, right median cingulate and paracingulate gyri, right posterior cingulate gyrus, left temporal pole: middle temporal gyrus); partial occipital lobe (left lingual gyrus, left paracentral lobule, right parahippocampal gyrus); and left thalamus. As the sgLasso method was based on the gLasso method for within-group selection, more overlap areas were obtained. In contrast, compared with the traditional lasso method, the sgLasso method had fewer overlapping regions, mainly concentrated in the partial parietal lobe (right central sulcus), left bilateral thalamus, partial frontal lobe (left superior frontal gyrus, medial), partial limbic lobe (right parahippocampal gyrus), and partial occipital lobe (left lingual gyrus). The results are shown in Figure 8.

**TABLE 3:** Brain regions that are significantly different computed by two different types of clustering coefficients.

**(A)** Brain regions that are significantly different computed by clustering coefficients defined on a single node.			
**ROIs**	***P*-value (HCC)**		
	**I**	**II**	**III**

**Lasso**			
Left supramarginal gyrus	**0.048**	0.214	0.118
Left rolandic operculum	0.118	0.118	**0.007**
Right rolandic operculum	0.303	0.094	**0.045**
Left superior frontal gyrus, medial	0.207	0.055	**0.007**
Right parahippocampal gyrus	0.638	**0.015**	**0.005**
Left thalamus	0.294	**0.049**	0.252
Left putamen	0.214	0.122	**0.047**
Right middle frontal gyrus	**0.019**	0.157	0.169
Left lingual gyrus	**0.017**	0.260	0.109
Right inferior occipital gyrus	0.060	**0.039**	**0.045**
Right fusiform gyrus	0.792	**0.047**	0.612
Right Paracentral lobule	0.393	**0.049**	0.090
Left middle temporal gyrus	0.804	**0.037**	0.181
**gLasso**			
Left inferior frontal gyrus, triangular part	**0.007**	0.968	0.063
Left inferior frontal gyrus, orbital part	**0.017**	0.817	**0.007**
Right rolandic operculum	0.265	0.991	**0.003**
Left median cingulate and paracingulate gyri	**0.038**	0.461	**0.005**
Right median cingulate and paracingulate gyri	**0.012**	0.201	**0.001**
Right posterior cingulate gyrus	0.303	0.341	**0.001**
Right hippocampus	**0.001**	0.058	**0.017**
Right parahippocampal gyrus	**0.006**	0.586	**0.016**
Right lingual gyrus	0.351	**0.006**	0.665
Right angular gyrus	**0.004**	**0.045**	0.080
Left precuneus	0.252	0.322	**0.005**
Left Paracentral lobule	0.094	0.147	**0.009**
Right Paracentral lobule	0.252	0.586	**0.002**
Left thalamus	0.087	**0.002**	0.404
**sgLasso**			
Right rolandic operculum	0.332	**0.043**	0.244
Right supplementary motor area	**0.003**	**0.033**	0.158
Left superior frontal gyrus, medial	0.142	0.404	**0.012**
Left median cingulate and paracingulate gyri	0.084	**0.012**	**0.010**
Right median cingulate and paracingulate gyri	0.229	**0.043**	**0.038**
Right posterior cingulate gyrus	0.164	**0.013**	**0.045**
Right parahippocampal gyrus	**0.045**	0.294	0.586
Left lingual gyrus	0.164	0.497	**0.005**
Left superior occipital gyrus	0.114	0.127	**0.032**
Left paracentral lobule	**0.032**	**0.014**	**0.023**
Left thalamus	**0.008**	0.351	0.208
Right thalamus	**0.025**	0.485	0.181
Left temporal pole: superior temporal gyrus	**0.019**	**0.001**	**0.016**

**(B)** Brain regions that are significantly different computed by clustering coefficients defined on a pair of nodes.					
**ROIs**	***P*-value (COMHCC)**				
	**I**	**II**	**III**	**IV**	**V**

**Lasso**					
Left precentral gyrus	0.229	**0.019**	**0.029**	0.2365	0.599
Right precentral gyrus	**0.033**	0.102	0.098	0.449	**0.047**
Left superior frontal gyrus, dorsolateral	0.201	0.612	0.331	0.461	**0.014**
Right middle frontal gyrus, orbital part	0.077	0.222	0.341	0.098	**0.003**
Left rolandic operculum	0.106	0.341	0.077	**0.016**	0.415
Left superior frontal gyrus, medial orbital	0.020	0.169	0.704	0.743	0.147
Right parahippocampal gyrus	0.294	0.164	0.449	**0.019**	0.900
Left amygdala	**0.004**	0.106	**0.025**	0.222	**0.033**
Left inferior occipital gyrus	0.152	**0.023**	**0.002**	0.839	0.244
Right inferior occipital gyrus	0.986	**0.017**	**0.045**	0.351	**0.041**
Right postcentral gyrus	0.252	**0.047**	0.215	0.900	0.188
Right angular gyrus	**0.018**	**0.023**	0.201	0.756	0.091
Right middle temporal gyrus	0.201	**0.006**	0.030	0.252	0.237
**gLasso**					
Left precentral gyrus	**0.011**	**0.009**	**0.003**	**0.011**	0.968
Left inferior frontal gyrus, orbital part	0.066	**0.015**	**0.030**	0.073	0.426
Left median cingulate and paracingulate gyri	**0.049**	0.123	0.053	0.087	0.181
Right median cingulate and paracingulate gyri	**0.041**	**0.011**	**0.017**	**0.032**	0.181
Right parahippocampal gyrus	0.056	0.142	**0.047**	0.052	0.268
Left cuneus	0.094	**0.036**	**0.009**	**0.025**	0.473
Left lingual gyrus	0.485	**0.013**	0.063	0.061	0.497
Right lingual gyrus	0.612	**0.025**	0.063	0.372	0.215
Left superior parietal gyrus	**0.004**	**0.032**	**0.026**	**0.049**	0.056
Left precuneus	0.252	0.158	0.142	**0.047**	0.312
Right superior temporal gyrus	**0.049**	0.147	**0.023**	**0.029**	0.121
Left temporal pole: middle temporal gyrus	0.151	**0.011**	0.098	0.158	0.910
**sgLasso**					
Left middle frontal gyrus	**0.035**	0.063	0.152	0.188	0.871
Right middle frontal gyrus	**0.041**	0.181	**0.043**	**0.035**	**0.020**
Left inferior frontal gyrus, orbital part	0.215	0.053	0.062	**0.033**	0.158
Right rolandic operculum	0.134	0.076	0.245	**0.042**	0.253
Right olfactory cortex	**0.047**	0.060	0.510	0.691	0.599
Right posterior cingulate gyrus	**0.018**	0.382	0.122	0.208	0.438
Right calcarine fissure and surrounding cortex	**0.029**	0.066	0.118	0.393	0.839
Left cuneus	0.098	0.084	0.313	0.828	0.029
Left thalamus	0.175	**0.032**	**0.038**	0.071	**0.021**
Right thalamus	0.181	**0.014**	**0.026**	0.817	**0.002**
Left temporal pole: middle temporal gyrus	**0.004**	**0.026**	**0.025**	0.118	0.573

*The bold values indicate the significant difference at p < 0.05.*

### Classification Performance

Classification performance was evaluated by measuring accuracy (ratio of correctly distinguished subjects), sensitivity (ratio of correctly distinguished patients), specificity (ratio of correctly distinguished normal persons), and balanced accuracy (BAC). Additionally, BAC is defined as the mean of sensitivity and specificity to avoid the expansion performance of unbalanced data sets (Velez et al., 2007).

We assessed the classification performance based on these three hypernetwork classification methods and compared them with traditional connectivity network (TCN) methods. The TCN method uses Pearson’s correlation to construct the functional brain network under a sparsity of 5–40%. The basic local metrics including degree, betweenness centrality, and node efficiency were calculated for all the subjects, and the area under the curve (AUC) value of each metric was computed to characterize the integrity properties of the index in the complete sparsity space. Then, the K-S non-parametric permutation test was done to select the local properties with significant intergroup differences as the classification feature. The classification results of these methods are summarized in Table 4.

**TABLE 4:** Classification performance of the three methods.

Methods	Research	Accuracy (%)	Sensitivity (%)	Specificity (%)	BAC (%)
TCN	Pearson-network	71.00	79	64	71.50
Lasso-based	Cluster coefficient based on single node	83.33	84.21	82.14	83.17
	Cluster coefficient based on pairs of nodes	74.24	78.95	67.86	73.41
	Multi-feature	84.85	89.47	78.57	84.02
gLasso-based	Cluster coefficient based on single node	80.30 ± 0.75	83.94 ± 0.82	75.35 ± 0.91	79.65
	Cluster coefficient based on pairs of nodes	75.76 ± 0.61	79.74 ± 0.63	70.35 ± 0.97	75.05
	Multi-feature	81.74 ± 0.69	84.74 ± 0.77	77.68 ± 0.91	81.21
sgLasso-based	Cluster coefficient based on single node	84.85 ± 0.75	87.89 ± 0.87	80.71 ± 0.99	84.30
	Cluster coefficient based on pairs of nodes	77.27 ± 0.82	80.79 ± 0.86	72.50 ± 1.13	76.65
	Multi-feature	87.12 ± 0.49	90.13 ± 0.47	83.03 ± 0.96	86.58

*TCN, traditional connectivity network method; lasso-based, hypernetwork based on lasso method; gLasso-based, hypernetwork based on gLasso method; sgLasso-based, hypernetwork based on sgLasso method; BAC, balanced accuracy; Multi-feature, the fusion feature between cluster coefficient based on single node and pairs of nodes.*

**FIGURE 9:**
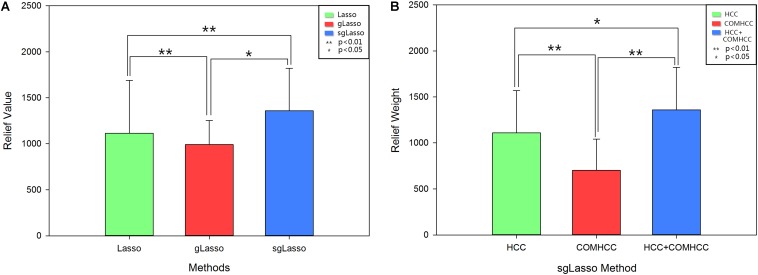
The ReliefF weight of brain regions feature. **(A)** The ReliefF weight of the corresponding feature of the divergence brain regions obtained by different methods. The *Y*-axis represents the ReliefF weight, and the *X*-axis indicates different methods used to construct the hypernetworks. Lasso denotes the ReliefF weight of the corresponding feature of the differential brain regions using the lasso method. gLasso denotes the ReliefF weight of the corresponding feature of the differential brain regions based on the gLasso method. SgLasso denotes the ReliefF weight of the corresponding feature of the differential brain regions calculated by the sgLasso method. **(B)** The ReliefF weight acquired by different feature extraction ways in the sgLasso method. HCC indicates the ReliefF weight obtained by HCC indicator features. COMHCC indicates the ReliefF weight obtained by COMHCC indicator features. HCC+COMHCC indicates the ReliefF weight by combining HCC indicators feature and COMHCC indicator features. Besides, ^∗∗^represents the *P* values obtained by non-parametric permutation test being less than 0.05, and ^∗^represents *P* values obtained by non-parametric permutation test being less than 0.01.

To compare the extent of the selected features of the three methods (the degree of contribution to the classification), the ReliefF algorithm was used to measure the classification weights of the corresponding different brain regions obtained by all three methods. The results are shown in Figure 9A, which identify that the weights of the features based on the sgLasso method are higher than the other two methods. Moreover, the sgLasso-based method showed the highest classification accuracy in the current study; hence, in the sgLasso method, we adopted the ReliefF algorithm to compute the corresponding feature weight based on the single node clustering coefficient feature, the mutual clustering coefficient feature, and the multi-features of the sgLasso method, respectively. The results indicate that the classification weights obtained by the multiple features are higher than the classification weights of the single features (Figure 9B).

## Discussion

Network construction is critical in the classification of brain networks based on hypergraph. Hypernetwork construction methods have been proposed in existing research, while some related brain regions cannot be selected in the construction of hyperedges owing to group structure problems among brain regions. In the lasso-based hypernetwork construction method proposed by Jie et al. (2016), the optimization objective function for solving the sparse linear regression model includes the loss function and the l_1_ norm penalty term. This penalty performs continuous compression and variable selection to render the network sparse. Considering the group structure problem, Guo et al. (2018) introduced the elastic net method and group lasso method to create a hypernetwork. The elastic net method used the l_1_, l_2_ penalty terms to make the model automatically select related variable groups; however, this does not generally mean that highly related variables belong to the active set in the group (Sjöstrand et al., 2018). The group lasso method employed the l_2,1_ norm to select variables on the basis of the predefined variable group (Yuan and Lin, 2006; Friedman et al., 2010), but it only carried out a selection of variables at the group level. Here, this study extended this research and proposed a new hypernetwork construction method based on the sgLasso method. In this method, the l_1_, l_2_ norm penalty term was introduced, i.e., its penalty was mixed into the lasso and group lasso penalty to perform the groupwise selection and intragroup variable selection. This is the bi-level selection that can be selected at the group level or at the level of individual covariates, which is different from the group level selection. In other words, using this method we could select not only important groups but also important variables within these important groups.

Hypernetworks are differences based on the three methods. Analysis of hyperedges showed a similar distribution between the MDD and NC groups. The hyperedge degree range based on the lasso method was distributed in the range of 2–13, and most of the hyperedges contained fewer nodes in the range of 2–7, being relatively tight. In the gLasso method, the range of hyperedge degree was distributed in 2–19, in that most of the hyperedges contained more nodes in the range of 8–19, being relatively loose. The edge degree in the sgLasso method mostly lay in 2–13. But it does not show a stronger ratio in the range of 2–7 (about 3/4 in MDD and NC group); that is, not most of the hyperedges connect a small number of nodes, but a considerable part of the hyperedge connects multiple nodes, which shows the network is intermediary. When building a hyperedge, the lasso method can only be used to select one of the brain regions in which a group structure exists. The gLasso method considers that all brain regions in the group are related when one brain region in the group is selected. However, the proposed method (sgLasso method) selects some brain regions associated with the group structure, mixing the lasso with the gLasso penalty term. Therefore, hypernetworks exit differences among three methods, in which the lasso method is the strictest; gLasso, the most lenient; and the sgLasso, moderate.

In addition, there were topological differences among the three methods of network construction with respect to the analysis of two different types of average clustering coefficients (mean HCC^1^-HCC^3^, mean COMHCC^1^-COMHCC^5^), regardless of the MDD or NC group. In the HCC indicator statistics, all statistical analysis showed that there was a significant difference between the average HCC^1^ and HCC^2^ based on the three hypernetwork construction methods. Significant differences were observed in the NC group in the lasso-based method and the other two methods, only no significant difference (*p* > 0.05 FDR corrected, *q* = 0.05) was found only in the gLasso and sgLasso methods in the mean HCC^3^. Meanwhile, statistical analysis showed significant differences in the average COMHCC^1^, COMHCC^2^, and COMHCC^4^ in the COMHCC properties. For the mean COMHCC^3^ and COMHCC^5^, there was no significant difference (*p* > 0.05 FDR corrected, *q* = 0.05) only in the sgLasso-based and gLasso-based approaches in the MDD group. Therefore, the results of both sets of indicators state that there are topological differences in the hypernetwork construction of these three methods.

Furthermore, we did a correlation analysis of indicators. The properties of all subjects in both the MDD and NC groups were averaged for every brain region. The linear regression analysis was performed based on the sgLasso method and traditional methods for the two sets of indicators, respectively. It was also observed that the sgLasso method was significantly correlated with the gLasso method (HCC indicator: Adj_Rsqr = 0.727 [NC group], Adj_Rsqr = 0.708 [MDD group]; COMHCC indicator: Adj_Rsqr = 0.864 [NC group], Adj_Rsqr = 0.832 [MDD group]), and the difference was larger than those obtained with the traditional lasso method (HCC indicator: Adj_Rsqr = 0.512 [NC group], Adj_Rsqr = 0.516 [MDD group]; COMHCC indicator: Adj_Rsqr = 0.523 [NC group], Adj_Rsqr = 0.491 [MDD group]). The potential reason is mainly because the sgLasso method selects variables from group level to groupwise, selecting important groups and further selecting important variables from within the group. This conclusion has also been verified in the analysis of significant difference regions. Upon comparison, hypernetwork topology by the sgLasso method was similar to the gLasso method but showed a difference by the lasso method. Meanwhile, hypernetwork differences were proved to exit in three methods, where the hypernetwork using the lasso method was the strictest; the group lasso, most lenient; and the sgLasso method, moderate. The potential reasons are likely the existence of the group structure and different degree of resolution about the group structure that led to this phenomenon. Besides, it is also proven that the constructed hypernetwork is not necessarily the best when only the group structure is selected. But if the group structure is appropriately extended, a relatively efficient hypernetwork topology can be obtained.

The significant difference regions obtained by statistical analysis are not the same for all three methods. There are more overlaps between the sgLasso and gLasso methods in comparison. The main reason is, like the gLasso method, groups needed to be divided before executing the sgLasso method, and then selected important groups and further selected important variables from within the group. Thus, overlapping regions are more in these two methods and less with the traditional lasso method. Moreover, three methods had fewer overlapping regions obtained from the two groups of clustering coefficients. This explained that biomarkers related to disease pathology were obtained more comprehensively. In conclusion, from the perspectives of edge distribution, topological data analysis, and significant difference regions, the article proved that the three networks are different and the network structures of sgLasso and gLasso are similar and different from lasso. The network created by the lasso method is strict, the network created by the gLasso method is relatively loose, and the network created by the sgLasso method is relatively moderate.

The best classification performance can be obtained in the sgLasso method. Therefore, we analyzed the abnormal brain region obtained by this method. After constructing the hypernetwork, different abnormal brain regions (HCC properties and COMHCC properties, including overlapping regions) were obtained by statistical calculation methods for two different types of clustering coefficients, including the partial parietal lobe (right central sulcus); right supplementary motor area; bilateral thalamus; partial frontal lobe (left superior frontal gyrus, medial, middle frontal gyrus, left inferior frontal gyrus, orbital part); partial limbic lobe (median cingulate and paracingulate gyri, right parahippocampal gyrus, right posterior cingulate gyrus, right olfactory cortex, right calcarine fissure, and surrounding cortex); partial occipital lobe (left cuneus, left lingual gyrus, left superior occipital gyrus, left paracentral lobule); and partial temporal lobe (left temporal pole: superior temporal gyrus, left temporal pole: middle temporal gyrus). These brain regions are consistent with the results mentioned in some of the previous literature (see Supplementary Table S1).

Three hypernetwork construction methods and correlation-based methods were applied to 38 patients with MDD and 28 NC subjects for classification. The results showed that the hypergraph-based brain network classification method can significantly improve classification performance. Moreover, the proposed method based on the sgLasso method showed the best classification performance of 87.12% when the parameter λ_2_ was set as 0.4. The classification results obtained by the sgLasso-based hypernetwork construction method were better than those obtained using the Lasso and gLasso methods, the potential reason being that it can perform bi-level selection; that is, both group level variables and groupwise variables can be selected. While the classification performance based on the lasso method was lower than the sgLasso method, whose underlying reason is perhaps that it can only choose one of the brain regions in the group structure, and it does not matter which one it chooses; this, in turn, leads to a very strict network build by lasso causing it to lose some important connections. This result implies that a more suitable hypernetwork cannot be constructed without considering the existence of the group structure. Similarly, the gLasso-based hypernetwork construction method is not as good as the sgLasso and lasso methods. The potential reason is that it does not have the flexibility of within-group variable selection, i.e., the relevant groups are only selected so that the estimated coefficients are all zero or all non-zero within each group, which causes the network built by gLasso to be very loose and lenient, likely adding some wrong connections. This result expresses that when constructing the hypernetwork, the group information should be considered, but the entire group information cannot be forced to be used and proper expansion of the group structure may be useful.

Finally, the importance of the feature was evaluated by the ReliefF algorithm, which is a feature-weighing algorithm. It assigns different weights according to the correlation of each feature and category. The greater the weight of the feature, the stronger the classification ability of the feature and vice versa (Kira and Rendell, 1992). In this study, the ReliefF algorithm was used to calculate the classification weights of the features obtained by different methods. The results showed that the weights of the features obtained by the sgLasso method were significantly larger than the other two methods (Figure 9A). This result suggests that the proper hypernetwork cannot be created without considering the existence of the group structure and only the groupwise structure. If the group structure is properly extended–that is, if a moderately constructed constraint (sgLasso method) is used–a valid hypernetwork can be obtained resulting in more effective classification features. Yet, construction strategies that are too strict (lasso method) or lenient (gLasso method) cannot achieve satisfactory effects. Apart from this, sgLasso is taken as an example to verify the validity of the fusion feature. The clustering coefficient characteristics of a single node and pairs of nodes and the multi-features are evaluated by the ReliefF algorithm. The results indicated that the ReliefF weight of the multi-feature fusion method was significantly higher than the ReliefF weight of the single feature (Figure 9B). The potential reason is that the multi-feature method effectively combines two different sets of information–the clustering coefficient characteristics defined on single node and that defined on two nodes–which can more wholly express the interaction information among brain regions. This result suggests that the multi-feature method is more suitable for assessing the importance of features. In addition, power analysis was performed for evaluating if the samples size was enough (see Supplementary Table S2 and Supplementary Text S4).

## Influence of Parameter and Repeatability Verification

Hypernetworks are created based on sparse regression models with penalty terms. Sparse linear regression models can help categorize a brain region based on a linear combination of other brain regions. Essentially, from a mathematical point of view, the most basic operation of the model is still pairwise correlation, but this is determined by the method itself. In addition, a penalty is added into the model, which forces some insignificant connections to be 0, such that a few brain regions are retained to interact with the selected brain region. Then, based on each subject, a few of the brain regions and a given brain region generated a hyperedge in a specific sparsity (that is, by fixing λ value, given vertex and all non-zero elements in the weight vector $\alpha_{m}^{n}$ formed a hyperedge) and all hyperedges consisted of a functional hypernetwork. Multivariate expression was performed in this way to represent the interaction between multiple brain regions in a brain function hypernetwork topology. Based on the original research and considering the group structure problem, we proposed to create a hypernetwork based on the sgLasso sparse regression models to obtain more effective biomarkers to more accurately diagnose brain diseases.

The classification performance of the classification method proposed in this paper depended on the selection of some parameters, such as the number of clusters *k*, hypernetwork construction model parameters λ_1_ and λ_2_, optimizing weight parameters *a*_i_. To address this issue, we carried out experiments based on the proposed (sgLasso) and the original (gLasso and lasso) brain hypernetwork.

### The Effect of the Number of Clusters *k*

**FIGURE 10:**
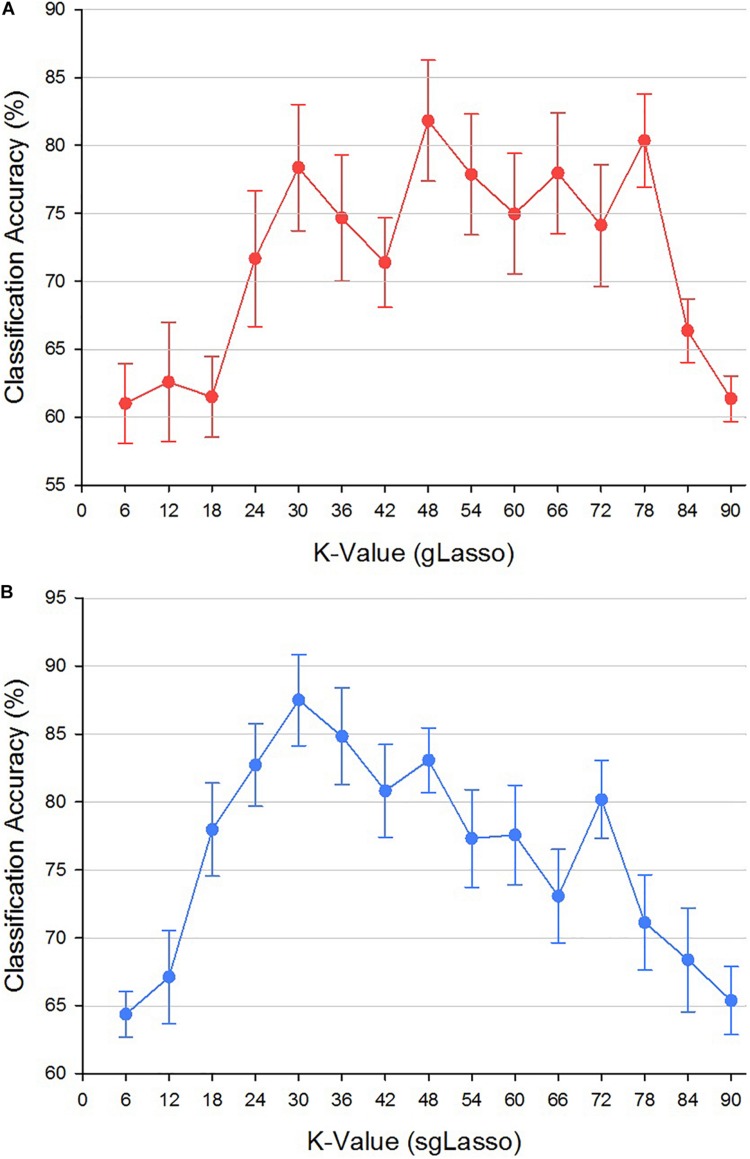
Classification accuracy of different *k* values based on gLasso and sgLasso methods. **(A)** Classification accuracy of different *k* values in gLasso method. **(B)** Classification accuracy of different *k* values in sgLasso method.

The parameter *k* is the number of groups clustered in the gLasso and sgLasso methods. A different *k* value will result in different functional network topologies and classification results. To explore the effect of *k* value on the classification performance, the variation range of *k* was set to 6–90 with the step size being 6. For each *k* value, we constructed a hypernetwork, extracted the features, and selected features based on gLasso and sgLasso methods. Then, the features of the two different types of indicators that are significantly different were classified using the SVM classifier based on multi-kernel learning, and the LOO was used to verify the classification effect. As random selection of the first initial seed point led to a difference in results, 50 experiments were performed for each method under each *k* value, and the average accuracy was selected as the final classification result. Figure 10 shows the experimental results of the two different methods. Figure 10A shows that the gLasso method had the highest classification accuracy of 81.74% when *k* = 48. Figure 10B shows that the sgLasso method had the highest accuracy of 87.12% when *k* = 30.

### The Effect of Regulation Parameters λ_1_ and λ_2_

Previous studies have demonstrated that parameter λ affected the topology of the hypernetwork. The sparsity and scale of the network were determined by the regularization parameter λ. If the λ value is too small, the network model constructed will be very rough and cause too much noise; on the contrary, if the λ value is too large, the network model will be comparatively sparse (Lv et al., 2015). It is worth noting that the sparsity of the network dominated by the λ value will affect the reliability of the network topology, especially modularity (Li and Wang, 2015). Besides, λ also had an impact on classification performance, which was sensitive to the final classification accuracy (Qiao et al., 2016). However, there is no gold standard on how to choose λ, so selecting the appropriate λ parameter is important for the construction and classification of the hypernetwork model. Some methods of selecting λ have also been used to optimize the network topology and classification performance in recent studies (Braun et al., 2012; Li and Wang, 2015; Qiao et al., 2016), but it was observed that it is difficult to achieve a highly reliable network structure by setting a single *λ.* Some studies have shown that the network can obtain relatively high reliability when λ is only 0.01 (very close to 0, which means that almost all nodes are connected at a hyperedge). In other cases, it performs modestly (Li and Wang, 2015). Therefore, multi-level λ is proposed (Jie et al., 2016). Unlike the single λ setting, the multi-level λ setting method sets a combination of several λ values, providing more network structure topology information than the setting of single λ method. The multi-level λ setting method can avoid any selection of a single λ setting method and drop the impact of a single network structure on low reliability. In the current study, the parameter λ_1_ is a regularization parameter of the l_1_ norm term, which controls within-group sparsity of the model, when the step size is set to 0.1. The parameter λ_2_ is a regularization parameter of the l_2_ norm term, which controls groupwise sparsity of the model, same as λ_1_, the step size is set to 0.1. Different choices of λ_1_ and λ_2_ will result in different solutions, which will cause different group variables being selected by the model, different group structures are arisen and then affect classification performance.

**FIGURE 11:**
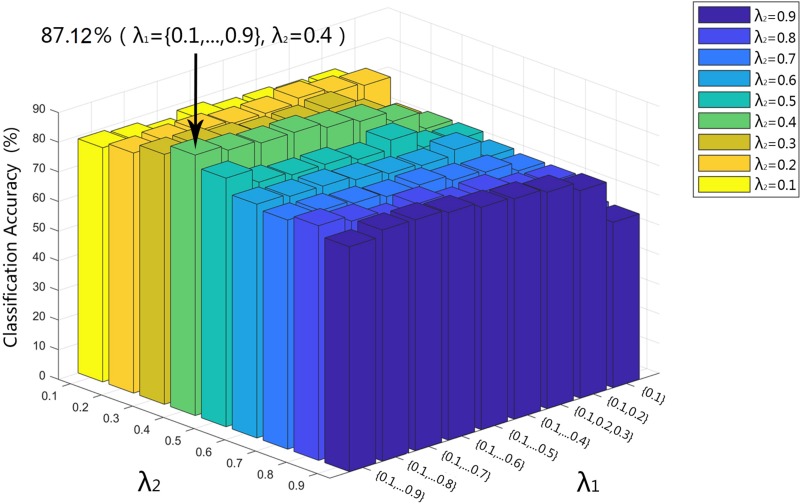
Classification accuracy of different regulation parameters (λ_1_, λ_2_).

For the setting of multi-level λ, it is important to determine how to obtain an optimized combination of λ. If every possibility is enumerated, the computational cost will be large. Therefore, a set of hyperedges were generated by having a fixed λ_2_ value and varying the λ_1_ value within a specific range in the construction of a hypernetwork based on the sgLasso methods. Meanwhile, to investigate the influence of λ_1_ and λ_2_ on the classification performance, λ_2_ was set to 0.1, 0.2, …, 0.9 respectively, and λ_1_ used a series of ascending order combinations, namely { 0.1 }, { 0.1, 0.2 }, { 0.1, 0.2, 0.3 }, …, { 0.1, 0.2, …, 0.9 }, to create different hypernetworks. In this study, the small λ values in the combinations were maintained as much as possible so that more nodes are connected in the hyperedge of the construction. This is because the hyperedge with many nodes can describe the potential information among several brain regions. Next, the features were extracted for the classification, which was then judged. The classification results are shown in Figure 11. The results show that the best accuracy is 87.12%, when λ_2_ = 0.4 and λ_1_ adopted { 0.1, 0.2, …, 0.9 } in the sgLasso method. When λ_1_ was {0.1}, the classification accuracy was lower than 60%, because some nodes were only included in one hyperedge. At this time, the denominator was zero in the HCC^3^ formula, so it was not effective to create the classification model.

### The Effect of Weight *a*_*i*_

In multi-kernel learning, the important step is the selection of weight parameters *a*_i_, which directly affects the way of data fusion and has a considerable effect on the classification performance. Here, a grid search method was adopted by obtaining the optimized weight in three methods, using the range from 0 to 1 with the step being 0.1. The accuracies of the lasso, gLasso, and sgLasso methods reached maximum values of 84.85, 81.74, and 87.12%, respectively, when the respective *a*_1_ and *a*_2_ values were 0.2 and 0.8, 0.3 and 0.7, and 0.2 and 0.8.

### Interpretability of SVM Classifier

Because the sgLasso method has the highest accuracy rate, the interpretability of SVM was discussed based on this method. LIME (Local Interpretable Model-agnostic Explanations) represents a local interpretation of agnostic models (Ribeiro et al., 2016). It is a tool that helps us understand and explain how complex machine learning models make decisions, which can explain multiple classifiers, including the SVM classifier. It mainly explains each sample individually. In this study, for each experiment, all samples were interpreted using LIME, so that features with higher weights are obtained, that is, features are obtained that contribute more to classification. After statistics, the conclusion was found that for each subject, LIME showed roughly the same features that make outstanding contributions to the diagnosis of the disease. These features are mainly HCC_123, HCC_40, HCC_229, HCC_77, COMHCC_285, HCC_227, HCC_78, HCC_83, COMHCC_87, where the corresponding brain regions of features are left median cingulate and paracingulate gyri, right parahippocampal gyrus, left superior occipital gyrus, left thalamus, Left inferior frontal gyrus, orbital part, left lingual gyrus, right thalamus, left temporal pole: superior temporal gyrus, and left temporal pole: middle temporal gyrus. Furthermore, via LIME analysis, the mean and confidence interval of the probability of the prediction results is listed for each subject in 50 experiments, see Supplementary Table S3.

### Repetitive Verification

To further validate the effectiveness of the above method, the Alzheimer’s Disease Neuroimaging Initiative (ADNI) data set^3^ was adopted to perform experiments in this study. Normal subjects and patients with Alzheimer’s disease were selected from the database, including 30 normal subjects and 29 Alzheimer’s patients. A preprocessing process that was similar to the above-described MDD data set was used, which comprised time layer correction, head motion correction, spatial normalization, linear dimensionality reduction, and band pass filtering and smoothing. The brain space was divided into 90 ROIs by the AAL template, and the average time series was extracted. Based on the average time series, three methods were used to construct the hypernetwork, and two different types of clustering coefficients were extracted as features. The non-parametric permutation test was used to select features, and the selected features were fused and classified by SVM. The classification performances of all three methods are summarized in Supplementary Table S4. The results showed that the sgLasso method achieved the best classification performance. This proved again that the proposed method is more advantageous and robust than the traditional hypernetwork construction method in describing brain function connections.

### Limitations

Our study has several limitations. First, the hypernetwork model parameters used in the experiment are the ratio for solving the sparse solutions. It is challenging to obtain the precise values due to technical limitations. Second, the random selection of initial seed points in the clustering and the difference of clusters *k* may have led to inaccurate functional network topology and classification results based on gLasso and sgLasso methods. For example, different clustering methods, such as a unified probabilistic model (Monti and Hyv Rinen, 2018), can be adopted for grouping, to ensure that a more stable hyperedge is established to further improve the hypernetwork. Finally, we used different templates for assigning brain regions to explore the impact of hypernetworks created by different templates on classification performance.

## Conclusion

The traditional brain function hypernetwork was created based on the lasso method. The main limitation of this method is that the group structure problem among the brain regions was not considered, and some correlated brain regions could not be selected. Therefore, the elastic net method and group lasso method were introduced to construct the hypernetwork in our previous study to solve this problem, and the result showed that the elastic net method obtained higher classification performance and could select highly correlated brain regions more accurately, but this does not generally mean that the active set (highly correlated variable) in the group is selected. Therefore, for solving the group structure problem, the previous method was extended and the sgLasso method introduced, to improve the hypernetwork creation in this study. At the same time, in the brain function hypernetwork, the previous research only involved the clustering coefficient of a single node as the feature extraction. However, according to several studies, the real network not only overlaps the neighbor nodes of a single vertex, but also has significant overlaps with neighborhood cohesiveness around the edges. Thus, in order to comprehensively assess disease performance and accurately identify biomarkers associated with pathology, clustering coefficients defined on two-node were introduced as feature extraction. Finally, the two sets of features were merged into a mixed kernel via multi-kernel learning for classification diagnosis.

Results of the analysis of the hyperedge, indicators of brain regions, and average indicators suggested that there are differences in the hypernetwork constructed by the three methods. The hypernetwork topology based on the gLasso method was similar to the sgLasso method, and conversely was different from the Lasso method. For network constraints, the lasso method was the most restrictive, gLasso method was the most relaxed, and the sgLasso method was moderate. This study analyzed the underlying causes and suggested that the existence of the group structure and degree of resolution of the group structure were responsible for the results obtained. Different constraints caused the change of classification accuracy, which showed that the classification performance based on the sgLasso method (87.12%) was better than the gLasso (81.74%) and lasso methods (84.85%). Moreover, evaluation of the different features of the two groups of clustering coefficients showed that the classification weights based on the sgLasso method are better than the gLasso and lasso methods, and the classification weights of multi-features are better than the classification weights of single features. This meant that a satisfactory effect cannot be obtained when there is no group structure (strict network construction) or only group level structure (loose network construction). If the group structure is appropriately extended (moderate network construction), efficient hypernetwork topology can be achieved.

## Data Availability Statement

The datasets generated for this study are available on request to the corresponding author.

## Ethics Statement

The studies involving human participants were reviewed and approved by the Medical Ethics Committee of Shanxi Province (reference number: 2012013). Written informed consent to participate in this study was provided by the participants’ legal guardian/next of kin.

## Author Contributions

YL was responsible for the study design and writing the manuscript. CS, PL, YZ, and GM performed the statistical analysis. YX provided and integrated the experimental data. HG and JC provided conception and design of the work. All authors approved the final version of the manuscript.

## Conflict of Interest

The authors declare that the research was conducted in the absence of any commercial or financial relationships that could be construed as a potential conflict of interest.

## Footnotes

1 http://www.fil.ion.ucl.ac.uk/spm

2 https://www.csie.ntu.edu.tw/~cjlin/libsvm/

3 http://adni.loni.usc.edu/

## Supplementary Material

FIGURE S1 Hypergraph. A hypergraph in which each hyperedge can connect more than two nodes. Here, the hypergraph consists of 8 nodes and 4 hyperedges. *V* = {v1,v2,v3,v4,v5,v6,v7,v8}, *E* = {e1,e2,e3,e4}, *e*1 = {v1,v2,v3}, *e*2 = {v3,v4,v5}, *e*3 = {v4,v5}, *e*4 = {v6,v7,v8}.

TABLE S1 Regions of interests chosen from other references on MDD.

TABLE S2 Comparison among Similar Researches.

TABLE S3 Probability to identify as a patient of all subjects.

TABLE S4 Classification performance of the three methods about AD dataset.

TEXT S1 Image Acquisition.

TEXT S2 Results of multiple linear regression analysis between network properties and confounding variables (cluster coefficients based on single node).

TEXT S3 Results of multiple linear regression analysis between network properties and confounding variables (cluster coefficients based on a pair of nodes).

TEXT S4 Power analysis for evaluating if the samples size was enough.

## References

[B1] Arthur D. Vassilvitskii S. (2007). “k-means++:the advantages of careful seeding,” in *Proceedings of the Eighteenth Acm-Siam Symposium on Discrete Algorithms, SODA*, New Orleans, LO.

[B2] Benjamini Y. Hochberg Y. (1995). Controlling the false discovery rate - a practical and powerful approach to multiple testing. *J. R. Statist. Soc.* 57 289–300. 10.1111/j.2517-6161.1995.tb02031.x

[B3] Braun U. Plichta M. M. Esslinger C. Sauer C. Haddad L. Grimm O. (2012). Test–retest reliability of resting-state connectivity network characteristics using fmri and graph theoretical measures. *Neuroimage* 59 1404–1412. 10.1016/j.neuroimage.2011.08.044 21888983

[B4] Bullmore E. Horwitz B. Honey G. Brammer M. Williams S. Sharma T. (2000). How good is good enough in path analysis of fMRI data? *Neuroimage* 11 289–301. 10.1006/nimg.2000.0544 10725185

[B5] Bullmore E. Sporns O. (2009). Complex brain networks: graph theoretical analysis of structural and functional systems. *Nat. Rev. Neurosci.* 10:186. 10.1038/nrn2575 19190637

[B6] Chen R. Herskovits E. H. (2007). Graphical-model-based multivariate analysis of functional magnetic resonance data. *Neuroimage* 35:635. 10.1016/j.neuroimage.2006.11.040 17258473 PMC2427148

[B7] Davison E. N. Schlesinger K. J. Bassett D. S. Lynall M. E. Miller M. B. Grafton S. T. (2015). Brain network adaptability across task states. *PLoS Comput. Biol.* 11:e1004029. 10.1371/journal.pcbi.1004029 25569227 PMC4287347

[B8] De Bie T. Tranchevent L. C. Van Oeffelen L. M. M. Moreau Y. (2007). Kernel-based data fusion for gene prioritization. *Bioinformatics* 23 i125–i132. 10.1093/bioinformatics/btm187 17646288

[B9] Dong P. Guo Y. Shen D. Wu G. (2015). Multi-atlas and multi-modal hippocampus segmentation for infant mr brain images by propagating anatomical labels on hypergraph. *Patch Based Techn. Med. Imaging* 9467 188–196. 10.1007/978-3-319-28194-0_23 30335869 PMC6166487

[B10] Estrada E. Rodr Guez-Vel Zquez J. A. (2006). Subgraph centrality and clustering in complex hyper-networks. *Phys. A* 364 581–594. 10.1016/j.physa.2005.12.002

[B11] Fasano G. Franceschini A. (1987). A multidimensional version of the Kolmogorov–smirnov test. *Month. Notic. R. Astron. Soci.* 50 9–20.

[B12] Fornito A. Zalesky A. Breakspear M. (2013). Graph analysis of the human connectome: promise, progress, and pitfalls. *Neuroimage* 80 426–444. 10.1016/j.neuroimage.2013.04.087 23643999

[B13] Friedman J. Hastie T. Tibshirani R. (2010). Regularization paths for generalized linear models via coordinate descent. *J. Statist. Softw.* 33 1–22. (accessed August 30, 2010). 20808728 PMC2929880

[B14] Gallagher S. R. Goldberg D. S. (2013). “Clustering coefficients in protein interaction hypernetworks,” in *Proceedings of the International Conference on Bioinformatics, Computational Biology and Biomedical Informatics*, (Wshington DC: ACM), 552–560.

[B15] Gao Y. Wee C.-Y. Kim M. GiannakopoulosMarie-Louise P. Haller M. S. (2015). MCI identification by joint learning on multiple MRI data. *Med. Image Comput. Comput. Assist. Interv.* 2015 78–85. 10.1007/978-3-319-24571-3_10 26942232 PMC4773025

[B16] Goldberg D. S. Roth F. P. (2003). Assessing experimentally derived interactions in a small world. *Proc. Natl. Acad. Sci.* 100:4372. 10.1073/pnas.0735871100 12676999 PMC404686

[B17] Gu S. Yang M. Medaglia J. D. Gur R. C. Gur R. E. Satterthwaite T. D. (2017). Functional hypergraph uncovers novel covariant structures over neurodevelopment. *Hum. Brain Map.* 38 3823–3835. 10.1002/hbm.23631 28493536 PMC6323637

[B18] Guo H. Li Y. Xu Y. Jin Y. Xiang J. Chen J. (2018). Resting-state brain functional hyper-network construction based on elastic net and group lasso methods. *Front. Neuroinform.* 12:25. 10.3389/fninf.2018.00025 29867426 PMC5962886

[B19] Huang J. Wang M. Shao W. Zhang D. (2018). Discovering network phenotype between genetic risk factors and disease status via diagnosis-aligned multi-modality regression method in Alzheimer’s disease. *Bioinformatics* 35 1948–1957. 10.1093/bioinformatics/bty911 30395195 PMC7963079

[B20] Huang J. Zhu Q. Hao X. Shi X. Gao S. Xu X. (2019). Identifying resting-state multifrequency biomarkers via tree-guided group sparse learning for schizophrenia classification. *IEEE J. Biomed. Health Inform.* 23 342–350. 10.1109/JBHI.2018.2796588 29994431

[B21] Huang S. Li J. Sun L. Ye J. Fleisher A. Wu T. (2010). Learning brain connectivity of Alzheimer’s disease by sparse inverse covariance estimation. *Neuroimage* 50 935–949. 10.1016/j.neuroimage.2009.12.120 20079441 PMC3068623

[B22] Jie B. Wee C. Y. Shen D. Zhang D. (2016). Hyper-connectivity of functional networks for brain disease diagnosis. *Med. Image Anal.* 32:84. 10.1016/j.media.2016.03.003 27060621 PMC5333488

[B23] Jie B. Zhang D. Gao W. Wang Q. Shen D. Wee C. Y. (2014). Integration of network topological and connectivity properties for neuroimaging classification. *IEEE Trans. Biomed. Eng.* 61 576–589. 10.1109/tbme.2013.2284195 24108708 PMC4106141

[B24] Kaiser R. H. Whitfieldgabrieli S. Dillon D. G. Goer F. Beltzer M. Minkel J. (2016). Dynamic resting-state functional connectivity in major depression. *Neuropsychopharmacology* 41 1822–1830. 26632990 10.1038/npp.2015.352 PMC4869051

[B25] Kaufmann M. Kreveld M. V. Speckmann B. (2016). “Subdivision drawings of hypergraphsin,” in *Proceedings of the International Symposium on Graph Drawing*, (New York, NY), 396–407. 10.1007/978-3-642-00219-9_39

[B26] Kira K. Rendell L. A. (1992). “The feature selection problem: traditional methods and a new algorithm,” in *Proceedings of the Tenth National Conference on Artificial Intelligence*, (New York, NY), 129–134.

[B27] Latapy M. Magnien C. Vecchio N. D. (2008). Basic notions for the analysis of large two-mode networks. *Soc. Netw.* 30 31–48. 10.1016/j.socnet.2007.04.006

[B28] Lee H. Lee D. S. Kang H. Kim B. N. Moo K. C. (2011). Sparse brain network recovery under compressed sensing. *IEEE Trans. Med. Imaging* 30 1154–1165. 10.1109/TMI.2011.2140380 21478072

[B29] Li X. Wang H. (2015). Identification of functional networks in resting state fMRI data using adaptive sparse representation and affinity propagation clustering. *Front. Neurosci.* 9:383. 10.3389/fnins.2015.00383 26528123 PMC4607787

[B30] Liu J. Ji S. Ye J. (2013). *Slep: Sparse Learning with Efficient Projections.* Arizona: Arizona State University.

[B31] Liu J. Ye J. (2010). “Moreau-Yosida regularization for grouped tree structure learning,” in *Advances in Neural Information Processing Systems 23: 24th Annual Conference on Neural Information Processing Systems*, (Tempe, AZ), 1459–1467.

[B32] Liu M. Zhang J. Yap P. T. Shen D. (2016). Diagnosis of alzheimer’s disease using view-aligned hypergraph learning with incomplete multi-modality data. *Med. Image Comput. Comput. Assist. Interv.* 2016 308–316. 10.1007/978-3-319-46720-7_36 28066842 PMC5207479

[B33] Liu X. Goncalves A. R. Cao P. Zhao D. Banerjee A. (2018). Modeling Alzheimer’s disease cognitive scores using multi-task sparse group lasso. *Comput. Med. Imaging Graph.* 66 100–114. 10.1016/j.compmedimag.2017.11.001 29602022

[B34] Lv J. Jiang X. Li X. Zhu D. Chen H. Zhang T. (2015). Sparse representation of whole-brain fMRI signals for identification of functional networks. *Med. Image Anal.* 20 112–134. 10.1016/j.media.2014.10.011 25476415

[B35] Lynall M. E. Bassett D. S. Kerwin R. McKenna P. J. Manfred K. Muller U. (2010). Functional connectivity and brain networks in schizophrenia. *J. Neurosci.* 30 9477–9487. 20631176 10.1523/JNEUROSCI.0333-10.2010 PMC2914251

[B36] Ma S. Song X. Huang J. (2007). Supervised group lasso with applications to microarray data analysis. *BMC Bioinform.* 8:60. 10.1186/1471-2105-8-60 17316436 PMC1821041

[B37] Mäkinen E. (1990). How to draw a hypergraph. *Int. J. Comput. Math.* 34 177–185. 10.1080/00207169008803875

[B38] Marrelec G. Horwitz B. Kim J. Pélégrini-Issac M. Benali H. Doyon J. (2007). Using partial correlation to enhance structural equation modeling of functional MRI data. *Magn. Resonan. Imaging* 25 1181–1189. 10.1016/j.mri.2007.02.012 17475433

[B39] Marrelec G. Krainik A. Duffau H. Pélégrini-Issac M. Lehéricy S. Doyon J. (2006). Partial correlation for functional brain interactivity investigation in functional MRI. *Neuroimage* 32 228–237. 10.1016/j.neuroimage.2005.12.057 16777436

[B40] Matsui H. (2018). Sparse group lasso for multiclass functional logistic regression models. *Commun. Statist. Simulat. Comput.* 48 1–14.

[B41] Meier L. Sara V. D. G. Hlmann P. (2008). The group lasso for logistic regression. *J. R. Statist. Soc.* 70 53–71. 10.1111/j.1467-9868.2007.00627.x

[B42] Montani F. Ince R. A. A. Senatore R. Arabzadeh E. Diamond M. E. Panzeri S. (2009). The impact of high-order interactions on the rate of synchronous discharge and information transmission in somatosensory cortex. *Philos. Trans. R. Soc. A Math. Physi. Eng. Sci.* 367 3297–3310. 10.1098/rsta.2009.0082 19620125

[B43] Monti R. P. Hyv Rinen A. (2018). A unified probabilistic model for learning latent factors and their connectivities from high-dimensional data. *arXiv.* [Preprint] Available at: https://arxiv.org/abs/1805.09567 (accessed May 24, 2018).

[B44] Nixon N. L. Liddle P. F. Nixon E. Worwood G. Liotti M. Palaniyappan L. (2018). Biological vulnerability to depression: linked structural and functional brain network findings. *Br. J. Psychiatry* 204 283–289. 10.1192/bjp.bp.113.129965 24357570

[B45] Ogutu J. O. Piepho H. P. (2014). Regularized group regression methods for genomic prediction: bridge, MCP, SCAD, group bridge, group lasso, sparse group lasso, group MCP and group SCAD. *BMC Proc.* 8:S7. 10.1186/1753-6561-8-S5-S7 25519521 PMC4195413

[B46] Ohiorhenuan I. E. Mechler F. Purpura K. P. Anita M. S. Hu Q. Victor J. D. (2010). Sparse coding and high-order correlations in fine-scale cortical networks. *Nature* 466 617–621. 10.1038/nature09178 20601940 PMC2912961

[B47] Park H. S. Jun C. H. (2009). A simple and fast algorithm for K-medoids clustering. *Expert Syst. Appl.* 36 3336–3341. 10.1016/j.eswa.2008.01.039

[B48] Pievani M. Agosta F. Filippi M. Frisoni G. (2011). Functional networks connectivity in patients with Alzheimer’s disease and mild cognitive impairment. *Brain* 7:170.

[B49] Qiao L. Zhang H. Kim M. Shen D. Teng S. H. Zhang L. (2016). Estimating functional brain networks by incorporating a modularity prior. *Neuroimage* 141 399–407. 10.1016/j.neuroimage.2016.07.058 27485752 PMC5338311

[B50] Ren P. Aleksić T. Wilson R. C. Hancock E. R. (2011). A polynomial characterization of hypergraphs using the Ihara zeta function. *Pattern Recogn.* 44 1941–1957. 10.1016/j.patcog.2010.06.011

[B51] Ribeiro M. T. Singh S. Guestrin C. (2016). “Why should I trust You?”: explaining the predictions of any classifier. *arXiv.org.* [Preprint], Available at: https://arxiv.org/abs/1602.04938 (accessed August 9, 2016).

[B52] Salvador R. Suckling J. Coleman M. R. Pickard J. D. Menon D. Bullmore E. (2005). Neurophysiological architecture of functional magnetic resonance images of human brain. *Cereb. Cortex* 15 1332–1342. 10.1093/cercor/bhi016 15635061

[B53] Santos G. S. Gireesh E. D. Plenz D. Nakahara H. (2010). Hierarchical interaction structure of neural activities in cortical slice cultures. *J. Neurosci.* 30:8720. 10.1523/JNEUROSCI.6141-09.2010 20592194 PMC3042275

[B54] Simon N. Friedman J. Hastie T. Robert T. (2013). A sparse-group lasso. *J. Computat. Graph. Statist.* 22 231–245.

[B55] Sjöstrand K. Clemmensen L. H. Larsen R. Einarsson G. Ersbøll B. (2018). Spasm: a MATLAB toolbox for sparse statistical modeling. *J. Statist. Softw.* 84 10.18637/jss.v084.i10

[B56] Sporns O. (2011). The human connectome: a complex network. *Ann. N. Y. Acad. Sci.* 1224 109–125. 10.1111/j.1749-6632.2010.05888.x 21251014

[B57] Sporns O. (2012). From simple graphs to the connectome: networks in neuroimaging. *Neuroimage* 62 881–886. 10.1016/j.neuroimage.2011.08.085 21964480

[B58] Tzourio-Mazoyer N. Landeau B. Papathanassiou D. Crivello F. Etard O. Delcroix N. (2002). Automated anatomical labeling of activations in SPM using a macroscopic anatomical parcellation of the MNI MRI single-subject brain. *Neuroimage* 15 273–289. 10.1006/nimg.2001.0978 11771995

[B59] Velez D. R. White B. C. Motsinger A. A. Bush W. S. Williams S. M. Moore J. H. (2007). A balanced accuracy function for epistasis modeling in imbalanced datasets using multifactor dimensionality reduction. *Genet. Epidemiol.* 31:306. 10.1002/gepi.20211 17323372

[B60] Wee C. Y. Yap P. T. Denny K. Browndyke J. N. Potter G. G. Welsh-Bohmer K. A. (2012). Resting-state multi-spectrum functional connectivity networks for identification of MCI patients. *PLoS One* 7:e37828. 10.1371/journal.pone.0037828 22666397 PMC3364275

[B61] Wee C. Y. Yap P. T. Zhang D. Wang L. Shen D. (2014). Group-constrained sparse fMRI connectivity modeling for mild cognitive impairment identification. *Brain Struct. Funct.* 219:641. 10.1007/s00429-013-0524-8 23468090 PMC3710527

[B62] Ye M. Yang T. Peng Q. Xu L. Jiang Q. Liu G. (2015). Changes of functional brain networks in major depressive disorder: a graph theoretical analysis of resting-state fMRI. *PLoS One* 10:e0133775. 10.1371/journal.pone.0133775 26327292 PMC4556670

[B63] Yu S. Yang H. Nakahara H. Santos G. S. Nikolić D. Plenz D. (2011). Higher-order interactions characterized in cortical activity. *J. Neurosci. Off.* 31 17514–17526. 10.1523/jneurosci.3127-11.2011 PMC6623824 22131413

[B64] Yuan M. Lin Y. (2006). Model selection and estimation in regression with grouped variables. *J. R. Statist. Soc.* 68 49–67. 10.1111/j.1467-9868.2005.00532.x

[B65] Zeng L. L. Shen H. Liu L. Wang L. Li B. Fang P. (2012). Identifying major depression using whole-brain functional connectivity: a multivariate pattern analysis. *Brain* 135:1498. 10.1093/brain/aws059 22418737

[B66] Zhang D. Wang Y. Zhou L. Yuan H. Shen D. (2011). Multimodal classification of Alzheimer’s disease and mild cognitive impairment. *Neuroimage* 55 856–867. 21236349 10.1016/j.neuroimage.2011.01.008 PMC3057360

[B67] Zhang J. Cheng W. Wang Z. G. Zhang Z. Lu W. Lu G. (2012). Pattern classification of large-scale functional brain networks: identification of informative neuroimaging markers for epilepsy. *PLoS One* 7:e36733. 10.1371/journal.pone.0036733 22615802 PMC3355144

[B68] Zhou D. Huang J. Sch B. (2006). “Learning with hypergraphs: clustering, classification, and embedding,” in *Proceedings of the 19th International Conference on Neural Information Processing Systems*, (Cambridge, MA: MIT Press), 1601–1608.

[B69] Zou H. Trevor H. (2005). Regularization and variable selection via the elastic net. *J. R. Statist. Soc.* 67 301–320. 10.1111/j.1467-9868.2005.00503.x

[B70] Zu C. Gao Y. Munsell B. Kim M. Peng Z. Cohen J. R. (2018). Identifying disease-related subnetwork connectome biomarkers by sparse hypergraph learning. *Brain Imaging Behav.* 13 879–892. 10.1007/s11682-018-9899-8 29948906 PMC6513717

