# Corrigendum: Hypernetwork Construction and Feature Fusion Analysis Based on Sparse Group Lasso Method on fMRI Dataset

**DOI:** 10.3389/fnins.2020.00243

**Published:** 2020-04-02

**Authors:** Yao Li, Chao Sun, Pengzu Li, Yunpeng Zhao, Godfred Kim Mensah, Yong Xu, Hao Guo, Junjie Chen

**Affiliations:** ^1^College of Information and Computer, Taiyuan University of Technology, Taiyuan, China; ^2^College of Arts, Taiyuan University of Technology, Taiyuan, China; ^3^Department of Psychiatry, First Hospital of Shanxi Medical University, Taiyuan, China

**Keywords:** hypernetwork, sparse group lasso, cluster coefficients based on pairs of nodes, multi-feature, classification, depression

In the original article, there was an error in the title. Instead of “Hypernetwork Construction and Feature Fusion Analysis Based on Sparse Group Lasso Method on Functional fMRI Dataset” it should be “Hypernetwork Construction and Feature Fusion Analysis Based on Sparse Group Lasso Method on fMRI Dataset”.

Additionally, in Equations (4) and (7) the parameters mentioned and its explanations were wrong. “n” should be superscript and not subscript. Also in Equation (4), “x” should not be in italics and “α” should be in italics. A correction has been made to the following sections:

The Materials and Methods section, subsection Construction of Hypernetwork, sub-subsection Sparse Linear Regression Model, paragraph 2:

“The average time series of *m*-th ROI for *n*-th subject, ${\text{x}}_{m}^{n}={\text{A}}_{m}^{n}\alpha_{m}^{n}+\tau_{m}^{n}$, can be viewed as a response vector, which can be estimated as a linear combination of time series of other ROIs. The sparse linear regression model is specifically expressed as follows:

(1) $$\begin{array}{r} {\text{x}}_{m}^{n}={\text{A}}_{m}^{n}\alpha_{m}^{n}+\tau_{m}^{n} \end{array}$$

where ${\text{x}}_{m}^{n}=\left[x_{m}^{n}\left(1\right);x_{m}^{n}\left(2\right);\ldots ;x_{m}^{n}\left(T\right)\right]$ refers to the average time series of the *m*-th ROI for *n*-th subjects, with T being the number of time points in the time series; ${\text{A}}_{m}^{n}=\left[{\text{x}}_{1}^{n},\ldots ,{\text{x}}_{m-1}^{n},0,{\text{x}}_{m+1}^{n}\ldots ,{\text{x}}_{M}^{n}\right]$ denotes the data matrix of the *m*-th ROI (all the average time series except for the *m*-th brain region, and the average time series of the *m*-th ROI being set to 0); $\alpha_{m}^{n}=\left[\alpha_{1}^{n},\ldots ,\alpha_{m-1}^{n},0,\alpha_{m+1}^{n}\ldots ,\alpha_{M}^{n}\right]$ denotes the coefficient vector that quantifies the degree of influence from the other ROI to the *m*-th ROI; and $\tau_{m}^{n}$ denotes a noise term, being Gaussian. The ROIs corresponding to the non-zero element in $\alpha_{m}^{n}$ are the ROIs interacting with the particular ROI; by contrast, the corresponding ROI of the zero element is conditionally independent with the *m*-th ROI.”

The Materials and Methods section, subsection Construction of Hypernetwork, Construction of Hypernetworks Based on the Sparse Group Lasso Method, paragraph 3:

“Similar to the gLasso method, clustering was adopted before creating the hyperedge, and then the sgLasso method was used to construct the hyperedge by solving the sparse linear regression model. The method is represented by the optimization objective function:

(2) $$\begin{array}{r} \underset{\alpha_{m}}{min}{\left||{\text{x}}_{m}^{n}-{\text{A}}_{m}^{n}\alpha_{m}^{n}|\right|}_{2}+\lambda_{1}{\left||\alpha_{m}^{n}|\right|}_{1}\text{+}\lambda_{2}\sum_{\text{i=}1}^{\text{k}}{\left||\alpha_{m}^{n}G_{i}|\right|}_{2} \end{array}$$

$\alpha_{m}^{n}$ is divided into k non-overlapping tree groups $\left(\alpha_{m}^{n}G_{1},\alpha_{m}^{n}G_{2},\ldots ,\alpha_{m}^{n}G_{k}\right)$by clustering, and *G*_*i*_ is a node with tree structure. λ_1_ and λ_2_ are regression parameters, with λ_1_ being used to adjust the sparsity of intra-groups to control the number of non-zero coefficients in non-zero groups, and λ_2_ being used to adjust group-level sparsity (Yuan and Lin, 2006; Friedman et al., 2010) to control the number of groups with non-zero coefficients. This model is a combination of traditional lasso and gLasso. The gLasso estimate is obtained when λ_1_ =0, and the lasso estimate is acquired when λ_2_ =0. It should be noted that the model looks somewhat similar to the elastic net model, but it is different because the l_2_ penalty is not differentiated at 0, so some groups are completely zeroed. However, in each non-zero group, it performs an elastic net fit (Simon et al., 2013). Like the gLasso method, a hypernetwork was constructed for each subject, where the ROI was regarded as the node, and the hyperedge comprised the m-th ROI and the ROIs corresponding to the zero elements in $\alpha_{m}^{n}$. For each ROI, a set of hyperedges were produced by fixing the λ_2_ value and varying the λ_1_value from 0.1 to 0.9 in increments of 0.1. Finally, a hypernetwork is a 90^*^810 matrix. In this experiment, the sgLasso method achieved the highest accuracy (87.12%) of all three models, when λ_2_was equal to 0.4. (see the Methodology Section about relative analysis).”

The authors apologize for these errors and state that this does not change the scientific conclusions of the article in any way. The original article has been updated.
