# Supplementary figures and images for: Hypernetwork Construction and Feature Fusion Analysis Based on Sparse Group Lasso Method on fMRI Dataset
Source: Front Neurosci. 2020 Feb 12;14:60. doi: 10.3389/fnins.2020.00060 (PMC7029661; doi:10.3389/fnins.2020.00060)

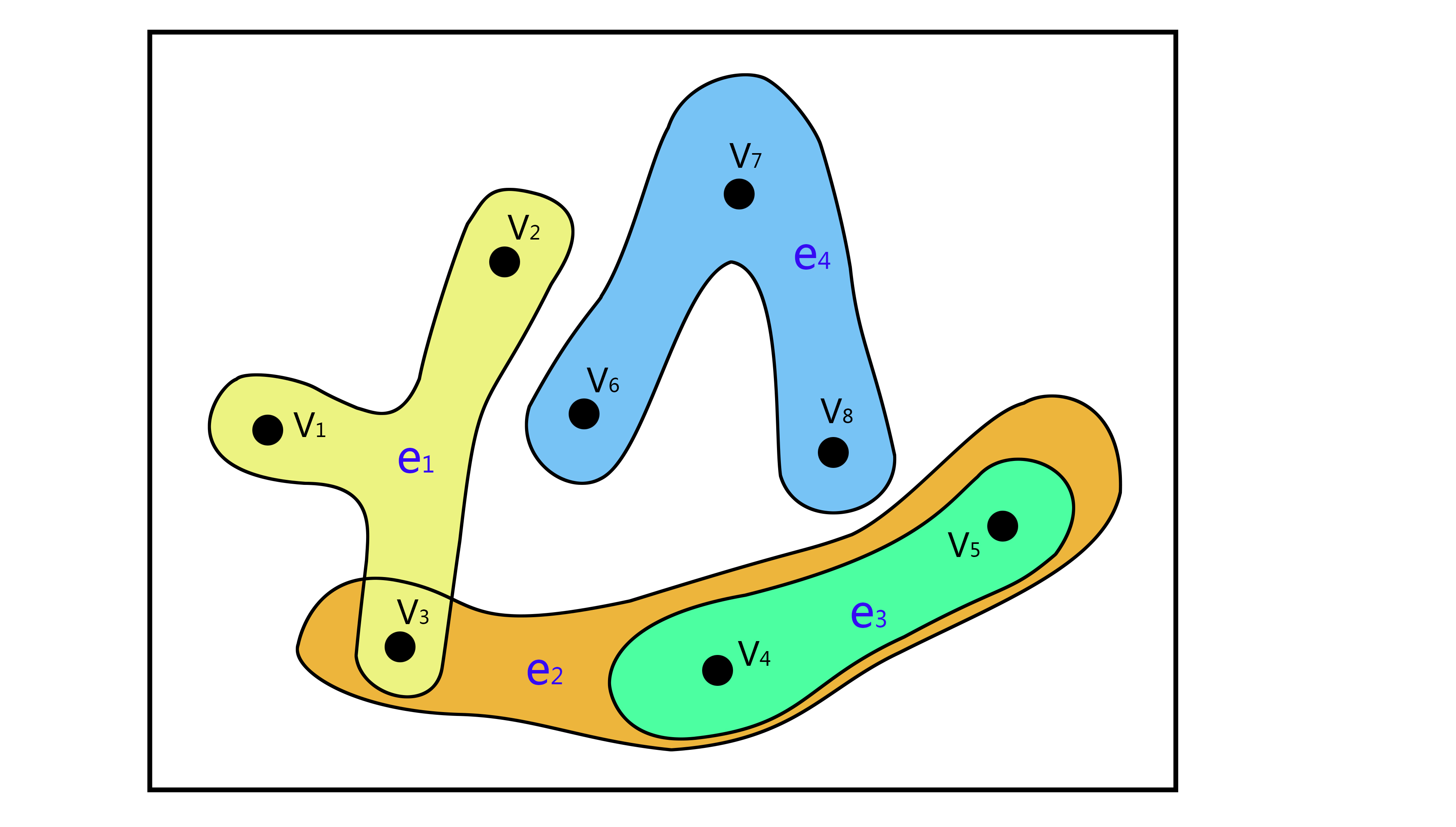

Supplement: FIGURE S1 — Hypergraph. A hypergraph in which each hyperedge can connect more than two nodes. Here, the hypergraph consists of 8 nodes and 4 hyperedges. V = {v1,v2,v3,v4,v5,v6,v7,v8}, E = {e1,e2,e3,e4}, e1 = {v1,v2,v3}, e2 = {v3,v4,v5}, e3 = {v4,v5}, e4 = {v6,v7,v8}. [file Image_1.JPEG]
