# Supplementary material for: Hypernetwork Construction and Feature Fusion Analysis Based on Sparse Group Lasso Method on fMRI Dataset
Source: Front Neurosci. 2020 Feb 12;14:60. doi: 10.3389/fnins.2020.00060 (PMC7029661; doi:10.3389/fnins.2020.00060)
Supplement: TABLE S1 — Regions of interests chosen from other references on MDD. [file Table_1.docx]

Supplemental Table S1. ROIs chosen from other references on MDD

| **ROIs** | **Citations** |  |
| --- | --- | --- |
| Right rolandic operculum | [Jingyu Zhu, et al., 2016] | [1] |
| Right supplementary motor area | [Feng Liu, Maorong Hu, et al., 2012] | [2] |
| Left superior frontal gyrus, medial | [Chenwang Jin, Chengge Gao, et al., 2011] | [3] |
| Median cingulate and paracingulate gyri | [Hao Guo, Xiaohua Cao, et al., 2012] | [4] |
|  | [Hao Guo, Pengpeng Yan, et al., 2018] | [5] |
| Right parahippocampal gyrus | [Lihua Qiu and Xiaoqi Huang, et al., 2014] | [6] |
| Right posterior cingulate gyrus | [Hao Guo, Xiaohua Cao, et al., 2012] | [4] |
| Left lingual gyrus | [Anton Lord, Dorothea Horn, et al., 2012] | [7] |
| Left superior occipital gyrus | [Feng Liu, Wenbin Guo, et al., 2013] | [8] |
| Left paracentral lobule | [Edmund T. Rolls, Wei Cheng, et al., 2018] | [9] |
| Thalamus | [Su Liu, Qizhu Wu, et al., 2011] | [10] |
| Middle frontal gyrus | [Hao Guo, Xiaohua Cao, et al., 2012] | [4] |
| Right olfactory cortex | [Hao Guo, Pengpeng Yan, et al., 2018] | [5] |
| Right calcarine fissure and surrounding cortex | [Junran Zhang, Jinhui Wang, et al., 2011] | [11] |
| Left cuneus | [Junran Zhang, Jinhui Wang, et al., 2011] | [11] |
| Left temporal pole: superior temporal gyrus | [Paul B. Fitzgerald, Angela R. Laird, et al., 2008] | [12] |
| Left temporal pole: middle temporal gyrus | [Lihua Qiu and Xiaoqi Huang, et al., 2014] | [6] |

# References

[1] ZHU J Y, SHEN X Y, QIN J L, et al. Altered Anatomical Modular Organization of Brain Networks in Patients with Major Depressive Disorder[C]// The International Conference on Biological Sciences and Technology. 2016.

[2] LIU F, HU M, WANG S, et al., Abnormal regional spontaneous neural activity in first-episode, treatment-naive patients with late-life depression: A resting-state fMRI study[J]*.* Progress in neuro-psychopharmacology & biological psychiatry, 2012. 39(2): 326-31.

[3] JIN C, GAO C, CHEN C, et al., A preliminary study of the dysregulation of the resting networks in first-episode medication-naive adolescent depression[J]*.* Neuroscience Letters, 2011. 503(2): 105-109.

[4] GUO H, CAO X, LIU Z, et al., Machine learning classifier using abnormal brain network topological metrics in major depressive disorder[J]*.* Neuroreport, 2012. 23(17): 1006-11.

[5] GUO H, YAN P, CHENG C, et al., fMRI classification method with multiple feature fusion based on minimum spanning tree analysis[J]*.* Psychiatry research. Neuroimaging, 2018. 277: 14-27.

[6] QIU L, HUANG X, ZHANG J, et al., Characterization of major depressive disorder using a multiparametric classification approach based on high resolution structural images[J]*.* Journal of Psychiatry & Neuroscience Jpn, 2014. 39(2): 78-86.

[7] LORD A, HORN D, BREAKSPEAR M, et al., Changes in community structure of resting state functional connectivity in unipolar depression[J]*.* PLoS One, 2012. 7(8): e41282.

[8] LIU F, GUO W, LIU L, et al., Abnormal amplitude low-frequency oscillations in medication-naive, first-episode patients with major depressive disorder: A resting-state fMRI study[J]*.* Journal of Affective Disorders, 2013. 146(3): 401-406.

[9] ROLLS E T, CHENG W, GILSON M, et al., Effective Connectivity in Depression[J]*.* Biological Psychiatry: Cognitive Neuroscience and Neuroimaging, 2018. 3(2): 187-197.

[10] LUI S, WU Q, QIU L, et al., Resting-State Functional Connectivity in Treatment-Resistant Depression[J]*.* American Journal of Psychiatry, 2011. 168(6): 642-648.

[11] ZHANG J, WANG J, WU Q, et al., Disrupted Brain Connectivity Networks in Drug-Naive, First-Episode Major Depressive Disorder[J]*.* Biological Psychiatry, 2011. 70(4): 334-342.

[12] FITZGERALD P B, LAIRD A R, MALLER J, et al., A meta-analytic study of changes in brain activation in depression[J]*.* Human Brain Mapping, 2008. 29(6): 683-695.
