# Supplementary material for: Hypernetwork Construction and Feature Fusion Analysis Based on Sparse Group Lasso Method on fMRI Dataset
Source: Front Neurosci. 2020 Feb 12;14:60. doi: 10.3389/fnins.2020.00060 (PMC7029661; doi:10.3389/fnins.2020.00060)
Supplement: TABLE S2 — Comparison among Similar Researches. [file Table_2.docx]

Supplemental Table S2. Comparison among Similar Researches

| **Paper[year]** | **Image Type** | **Disease/Groups** | **Patients/N** | **Patients/Age** | **Control/N** | **Control/Age** | **Node Definition** | **Feature Selection Method** | **Features** | **Machine Learning Method** | **Validation** |
| --- | --- | --- | --- | --- | --- | --- | --- | --- | --- | --- | --- |
| Robinson EC et al. [2010] | sMRI | Age (Young / /Seniors) | / | 20-30,59-90 | / | / | AAL(90) | / | Edge FA | MLDA | ACC=87,SEN=90,SPE=88,Bayeserr.=0.87 |
| Hui Shen et al. [2010] | fMRI | Schizophrenia | / | 19-30 | / | / | AAL(90) | / | ROI Signal Correlations | C-Means | ACC=92,AUC=0.96 |
| C.-Y. Wee et al. [2011] | sMRI | MCI | 10 | 74,/-8.6 | 17 | 74±8.6 | AAL(90) | SVM-RFE | Local CC | SVM | ACC=89, AUC=0.93 |
| Nico U. F. Dosenbach et al.[2011] | fMRI | Age Groups | / | 7 to 30 | , | 7 to 30 | Learned(210) | T-test(p<0.001) | ROI Signal Correlations | SVM | ACC=91,SEN=90,SPE=92 |
| Jonas Richiardi et al. [2011] | fMRI | Resting/Watching Movie | / | 18-36 | / | / | AAL(90) | T-test (p <0.05) | Multi-band Time Series | Polythetic Decision Trees | ACC=97 |
| Zhang J et al. [2011] | fMRI | MDD | 30 | 18–60 | 63 | 16–81 | AAL(90) | / | Small-world, Efficiency, and Nodal Centrality | / | / |
| Jin C et al.[2011] | fMRI | MDD | 16 | / | 16 | / | AAL(90) | / | Degree , BC, Efficiency | / | / |
| Wei Cheng1 et al. [2012] | fMRI | ADHD | 101 | 12.08 ± 2.05 | 143 | 11.43± 1.86 | AAL(90) | 1.T-tests(p < 0.008)  2.BWAS | ROI Signal Partial , Full Correlations | SVM | ACC=76,SEN=63,SPE=85 |
| Hai Li et al.[2012] | DTI | ASD | 10 | 7-14 | 10 | 7-14 | JHU-DTI-MNI atlas(46) | SVM-RFE | Edge Connectivity | SVM | ACC=100 |
| Soltanian-Zadeh et al. [2012] | fMRI | Rest/Attention | 19 | 20-30 | 19 | 20-30 | AAL(90) | Recursive feature ranking , WL Kernel | ROI Signal Correlations | SVM | ACC=100 |
| Jonas Richiardi et al. [2012] | fMRI | MS | / | 29-45 | / | / | AAL(90) | / | ROI Signal Correlations | Ensemble of Functional Trees | ACC=83,SEN=82,SPE=86 |
| Danesh Shahnazian et al. [2012] | fMRI | Rest/Attention | / | 20-30 | / | / | Conditional Granger Causality Analysis(CGCA)(24) | / | Bi-variate Granger Causality Network | SVM | ACC=100 |
| D. S. Bassett et al. [2012] | fMRI | Schizophrenia | 29 | 41.3 ± 9.3 | 29 | 41.1 ± 10.6 | AAL(90) | / | Largest Connected Component Size Curve | SVM | ACC=75,SEN=85,SPE=64 |
| C.-Y. Wee et al. [2012] | sMRI,fMRI | MCI | 10 | 64-83 | 17 | 64-83 | AAL(90) | T-test,Linear,p(0.004-0.01) Polynomial,RBF kernels | Structural and Functional Nodal CC | Multi-kernel SVM | ACC=96, SEN=100, SPE=94, AUC=95, YDI=94, BAC=95, |
| Lord AHorn D et al.[2012] ] | fMRI | MDD | 22 | / | 22 | / | AAL(90) | mRMR | PI, LE/GE, LE, Degree and BC | SVM | ACC =99 |
| Guo H et al. [2012] | fMRI | MDD | 38 | 17–54 | 28 | 17–51 | AAL(90) | T-test ( p<0.05) | Small-world, Efficiency, and Nodal Centrality | SVM | ACC =79.27 |
| Bai F et al.[2012] | DTI | RGD,aMCI | 35 | / | 30 | / | AAL(90) | / | Topological Metrics | / | / |
| Z.-W. Peng, et al. [2013] | fMRI | AD,MCI | 25 | 65-83 | 25 | 65-83 | AAL(90) | T-test ,RFE(p<0.005) | ROI Signal Correlations | SVM | ACC=92, AUC=90 |
| Biao Jie er al.[2013] | fMRI | MCI | 12 | 75.0±8.0 | 25 | 72.9±7.9 | AAL(90) | Linear-kernel-based,Graph Kernel | Local CC, Local Connectivity and Global | SVM | ACC=92 |
| C.-Y. Wee et al. [2013] | fMRI | MCI | 29 | 68-80 | 30 | 68-80 | AAL(90) | Fused multiple graphical lasso | Temporal Sliding Window Region Activations | SVM | ACC=90, BAC=79, SEN=76, SPE=83, AUC=0.79 |
| M. R. Arbabshirani et al. [2013] | fMRI | Schizophrenia | 28 | 36.5 ± 11.3 | 28 | 39.7 ± 10.1 | ROI (358) | 1.T-test (p<0.05);  2. Correlation-based Feature Selection (CFS) | ROI Signal Correlations | SVM | ACC=96,SEN=100,SPE=92,PPV=92,NPV=100 |
| K.Caeyenberghs et al.[2013] | sMRI,fMRI | TBI | 16 | / | 17 | / | ROI(22) | T-tests(p<0.05) | Degree, Efficiency, Betweenness centrality | Modality | ACC=61,SEN=43,SPE=77 |
| Tao H et al. [2013] | fMRI | MDD | 15 | / | 37 | / | AAL(90) | / | / | / | / |
| Singh M et al.[2013] | sMRI | MDD | 93 | 18–60 | 151 | 18–60 | AAL(90) | / | Clustering ,Path Length | / | / |
| Qin J et al.[2013] | DTI | MDD | 29 | 22–53 | 30 | 23–54 | AAL(90) | / | Network Metrics | / | / |
| D. Zhu et al. [2014] | fMRI,sMRI | MCI, Schizophrenia | 10 | / | 18 | / | DICCCOL(358) | Edge T-test, CFS(p<0.005) | ROI Signal Correlations | SVM | ACC=96 |
| Fei, Fei et al. [2014] | fMRI | MCI | 12 | 75.0±8.0 | 25 | 72.9±7.9 | AAL(90) | DSM , WL kernel | GSpan Frequent Subnetworks | Graph-kernel | ACC=97,AUC=0.96 |
| D. Zhu et al. [2014] | fMRI,sMRI | MCI | 10 | (55-84), (66-84) | 18 | (55-84), (66-84) | DICCCOL(359) | CFS | ROI Signal Correlations | CFS | ACC=100, 96 |
| Ghanbariet al.[2014] | sMRI | ASD, Age | 24 | 7.8-18.3 | 59 | 7.8-18.3 | ROI(79) | Built-into NMF | Edge Probability | NMF, Graph Embeddings | / |
| Jie, Biao et al.[2014] | fMRI | MCI | 12 | 75.0±8.0 | 25 | 72.9±7.9 | AAL(90) | 1.T-test(P [0.05 0.08 0.10 0.12 0.15])  2.RFE:Graph Kernel (Denoted as RFE-GK) | Local CC | SVM | ACC=92,BAC=94,SEN=100,SPE=88,AUC=94 |
| Biao Jie et al.[2014] | fMRI | MCI | 12 | / | 25 | / | AAL(90) | M2TFS | Local CC | SVM | ACC=95,SEN=92,SPE=96,AUC=0.96 |
| Pariyadath Vani et al.[2014] | fMRI | Smoking Status | 21 | 28-50 | 21 | 28-50 | ICA ,Clustering(56) | SVM-RFE | Correlations within and between Subnetwork Regions | SVM | ACC=79, PPV=83 |
| L. Wang et al. [2014] | fMRI | MCI | 12 | 65-83 | 25 | 65-83 | AAL(90) | T-test(p<0.05),REF, gspan | Local CC | SVM | ACC=97, AUC=0.92 |
| KAMIYA Kouhei et al. [2015] | DTI | TLE | 44 | 21-45 | 14 | 21-45 | ROI(83) | Sparse Linear Regression | Local Network Measures | SVM | ACC=90, AUC=0.97 |
| Khazaee, Ali et al.[2015] | fMRI | AD | 20 | 74.85 ± 4.50 | 20 | 75.05 ± 4.92 | AAL(90) | Fischer Score(p < 0.005) | Local , global network measures | SVM | ACC=100 |
| Bo-yong Park et al.[2015] | sMRI,fMRI | BMI | 60 | 29 | 60 | 29 | AAL(90) | T-test,Permutation Testing, Region Prior,Functional-Structural Correlation | Edge Fiber Density , fMRI Mean Nodal Degrees | PLSR | MAE=15%, RMS=5.3 |
| John R et al. [2015] | fMRI | Age and Clincal Risk(Low, High) | / | 6-12months | / | / | ROI(230) | T-test , Linear Kernel (p < 2.2251e-308) | ROI Signal Correlations | SVM | ACC=81,75,SEN=78,81,SPE=84,69 |
| Qiu et al. [2015] | fMRI | Age | / | 22-79 | / | / | / | / | ROI Signal Partial Correlations | Linear Regression | r=0.59,RMS=12.9 |
| Sacchet et al. [2015] | sMRI | MDD | 14 | 18-55 | / | / | Desikan–Killiany Atlas(68) | T-test (p <0.05) | Global Network Measures (9) | SVM | ACC=72,SEN=71,SPE=72 |
| Brent C. Munsell et al. [2015] | fMRI | TBI and AD | / | 70 | / | / | AAL(90) | / | ROI Signal Partial Correlations | SVM | ACC=82,SEN=40,SPE=98,PPV=86,NPV=81 |
| C. J. Brown et al. [2015] | sMRI | Low Motor Function | 146 | 24-32 | 22 | 27-4 | AAL(90) | PCA | FA | SVM | ACC=72,SEN=77,SP=69 |
| Ota K et al.[2015] | fMRI | MCI | 40 | / | 23 | / | AAL(90) | SVM-RFE | GM Density | SVM | ACC=77 SEN=97 SPEC=50 |
| L. Zhan et al. [2015] | sMRI,MRI | NC/MCI/ AD | AD：39 MCI：112 NC：51 | ADNI-2 | / | ADNI-2 | ROI(113) | High Order SVD | Edge Tract Counts | SLR | ACC=71, SEN=68, SPE=72, AUC=0.76 |
| Chung AW et al.[2016] | sMRI | Low Motor Function | 55 | 28.28±2.25 weeks | 233 | 30.01±2.23 weeks | AAL(90) | Heat kernel methodology | Heat Flow Features | Gaussian Naive Bayes | ACC=82,SEN=75,SPE=83,fs=79 |
| C.-Y. Wee et al. [2016] | fMRI | ASD | 45 | 7--15 | 47 | 7--15 | AAL(90) | Lasso | ROI Signal Correlations | SVM | ACC=71, SEN=80, SPE=61, |
| J. Galvis et al. [2016] | DWI | Parkinson’s Disease | 58 | / | 131 | / | FreeSurfer(129) | T-test(p<0.005) | ROI Signal Correlations | SVM | BAC=60 |
| D. Gellerup. Et al.[2016] | fMRI | Parkinson’s Disease | 24 | 60 | 21 | 61.9 | Power (264) | mRMR | ROI Signal Correlations | Proximal SVM Ensemble | ACC=0.84, SEN=0.73, SPE=93 |
| Jin Y et al.[2016] | sMRI | ASD | 40 | six-month | 40 | six-month | Atlas(90,203,403) | T-test(p < 0.001), LASSO | Edge FA, MD and TC, Multiple Scales | SVM | ACC=76,SEN=72,SPE=79,AUC=0.8 |
| Mitra J et al. [2016] | sMRI | TBI | 179 | / | 215 | / | AAL(90) | NBS Edge T-test (p < 0.002), PCA | Edge FA | Random Forest | ACC=68,SEN=80,SPE=46,PPV=68,NPV=69 |
| Christopher D. Smyser et al. [2016] | fMRI | Age | 50 | 23-29weeks | 50 | the first week | ICBM462(55) | T-test Filter(p<0.005) | ROI Signal Correlations | SVM | ACC=84,SEN=90,SPE=78 |
| J. Kawahara et al. [2016] | sMRI | Motor,Cognitive Function,Age | / | / | / | / | ROI(90) | T-tests(p<0.05) | Edge Tract Counts | BrainNetCNN | r=0.31,0.19, 0.86,MAE=10,10.5, 2.3 |
| G. Ball et al. [2016] | fMRI | Preterm /term, age | 26 | 23 ,6-48,0 | 105 | 37,1-46,2 | ICA(71) | Boruta algorithm((p<0.05) | ROI signal covariances | SVM,RBF,Linear Regression | BAC=80,AUC=0.92, MSE=8.9 |
| C. J. Brown et al. [2016] | sMRI | Low Motor,  Cognitive Function | 139/155 | / | 13/29 | / | AAL(90) | / | Edge Tract Counts | Non-negative linear Regression | ACC=71,AOC=14.3,r=0.44 |
| Biao Jie et al.[2016] | fMRI | MCI | 12 | 75±8 | 25 | 72.9±7.9 | AAL(90) | T-test (p<0.05) | Clustering Coefficients | SVM | ACC=82.9 SEN=83.9 SPE=81.6 |
| Hao Guo et al.[2017] | fMRI | AD | 38 | / | 28 | / | AAL(90) | T-test (p<0.05) | / | SVM | ACC=98.1 SEN=98.9 SPE=96.6 |
| Ali Khazaee et al. [2017] | fMRI | MCI and AD | AD(34) MCI(89) | 72.5 | 45 | 75.9 | AAL(90) | FSFS and Fisher Algorithm | Local and Global Graph | SVM | ACC=93.3 |
| Seyed Hani Hojjati et al. [2017] | rs-fMRI | MCI and AD | MCI-C(18) MCI-NC(62) | 73.6 | / | / | AAL(90) | T-test(p < 0.05) Fisher Score, Chi-square Score, Gini Score, and Kruskal-Wallis Test(p<0.05) | / | SVM | ACC=91.4 SEN=83.2 SPE=95 |
| De Marco et al. [2017] | rs-fMRI | MCI | 50(31/19) | 73.86 | 50(25/25) | 69.54 | AAL(90) | LDA and QDA | Blood Oxygen Level Dependent-Connectivity | SVM | ACC=90 |
| Ioannis Gallos et al. [2017] | rs-fMRI | Schizophrenia | 72 | 38.16 | 74 | 35.86 | Clustering(40-65) | PCA /T-test(p < 0.01) | / | SVM | ACC=63.5 |
| Chen, Xiaobo et al. [2017] | rs-fMRI | MCI | 54 | / | 54 | / | AAL(90) | T-test ( p < 0.05) and LASSO regression | RMS for Each Signal | SVM | ACC=78,SEN=77.7,SPE=79.6 |
| Alam et al. [2017] | MRI | MCI and AD | AD(89) MCI(102) | AD 77.3 MCI 77.36 | 102 | 76.05 | AAL(90) | KPCA , LDA | / | SVM | ACC=86.5 |
| Xiaofeng Zhu et al. [2017] | MRI | MCI and AD | AD(51) MCI(99) | / | 51 | / | AAL(90) | Sparse Multi-task Learning Framework | / | SVM | ACC=92 |
| Gurevich Pavel et al. [2017] | MRI | MCI and AD | AD(70) | 69.8 | non-AD(88) | 71.4 | / | PCA | / | SVM | ACC=82 |
| Alam Saruar et al. [2017] | MRI | AD | AD 86 | 77.3 | 86 | 76.05 | PCA | PCA | Principal Coefficients | SVM | ACC=92.6,SEN= 97.7,SPE=95.6 |
| Yu Zhang et al.[ 2017] | rs-fMRI | MCI | 53 | / | 59 | / | AAL(90) | Sparse Regression | Weighted Clustering Coefficients | SVM | ACC=83.8,SEN= 79.3,SPE=88.1 |
| Zhang Y et al.[2017] | rs-fMRI | eMCI | 13F/16M | / | 17F/13M | / | AAL(90) | Sparse Learning | Weighted-graph Local Clustering Coefficients | SVM | ACC=88.1,SEN=86,SPE= 90 |
| Yousra Asim et al.[2017] | sMRI,MRI | AD | 100 | 55-90 | 100 | 55-90 | AAL(90),LPBA40 | PCA | GM | SVM | ACC=94 SEN=95 |
| Y Li et al.[2017] | fMRI | MCI | 28 | / | 33 | / | AAL(90) | T-test (p<0.05) | Clustering Coefficients | SVM | ACC=86.9 SEN=82.1 SPE=90.9 |
| Guo Hao et al.[2017] | fMRI | MDD | 38 | / | 28 | / | AAL(90) | FSFS | Subgraph | SVM | ACC=88.9 SEN=91.7 SPE=85.6 |
| Hojjati et al. [2018] | sMRI,rs-fMRI | MCI | 18 | 73.6 ± 15.7 | 62 | 73.0 ± 16.3 | AAL(90) +Dosenbach Atlas | T-test(p<0.05) | / | SVM | ACC=91.4/ACC=89 |
| Bouts, Mark et al. [2018] | fMRI | AD | 41 | / | 40 | / | AAL(90) | T-test(p<0.05) | / | Elastic Net Regression | ACC=0.90,SEN=0.89,SPE=0.90 |
| Zhao Feng et al [2018] | RS-fMRI | ASD | 47 | 10.7 ± 2.28 | 40 | 11.22 ± 2.34 | AAL(90) | LASSO | ROI Signal Correlations | SVM | ACC=81 |
| Yang P et al.[2018] | fMRI | MCI | 24 | / | 23 | / | AAL(90) | Group Lasso | ROI Signal Covariances | SVM | ACC=93.6 SEN=91.6 SPE=95 |
| Chen Zu et al.[2018] | fMRI | AD | 50 | / | 50 | / | AAL(90) | T-test ( p<0.05) | Three-element Vector | SVM-STM | ACC=87 SEN=87 SPE=87 |
| NianyinZeng et al.[2018] | fMRI | AD | 92 | / | 92 | / | AAL(90) | PCA | ROI Signal Covariances | SDPSO-SVM | ACC=81.2 |
| SI Dimitriadis et al.[2018] | fMRI | AD | 100 | 74.9±5.6 | 100 | 73.1±8.2 | AAL(90) | KNIME Plugin K-Surfer | / | RF | ACC=61.9 |
