# Supplementary material for: Hypernetwork Construction and Feature Fusion Analysis Based on Sparse Group Lasso Method on fMRI Dataset
Source: Front Neurosci. 2020 Feb 12;14:60. doi: 10.3389/fnins.2020.00060 (PMC7029661; doi:10.3389/fnins.2020.00060)
Supplement: TABLE S3 — Probability to identify as a patient of all subjects. [file Table_3.docx]

Supplemental Material Table S3 Probability to identify as a patient of all subjects

| Subject serial number | Probability to identify as a patient | Confidence interval ($\pm$) |
| --- | --- | --- |
| 1 | 0.752183 | 0.039326 |
| 2 | 0.81073 | 0.045827 |
| 3 | 0.835703 | 0.040912 |
| 4 | 0.881392 | 0.046327 |
| 5 | 0.827074 | 0.036808 |
| 6 | 0.855276 | 0.052022 |
| 7 | 0.767741 | 0.058907 |
| 8 | 0.831016 | 0.069811 |
| 9 | 0.809612 | 0.058875 |
| 10 | 0.873228 | 0.056708 |
| 11 | 0.791094 | 0.042767 |
| 12 | 0.784297 | 0.080392 |
| 13 | 0.868092 | 0.054477 |
| 14 | 0.793034 | 0.060116 |
| 15 | 0.882441 | 0.060419 |
| 16 | 0.853881 | 0.057954 |
| 17 | 0.773354 | 0.057399 |
| 18 | 0.926127 | 0.033138 |
| 19 | 0.914265 | 0.045206 |
| 20 | 0.800765 | 0.04694 |
| 21 | 0.940888 | 0.026758 |
| 22 | 0.782091 | 0.056889 |
| 23 | 0.738376 | 0.035989 |
| 24 | 0.946166 | 0.034968 |
| 25 | 0.805643 | 0.047173 |
| 26 | 0.775676 | 0.036983 |
| 27 | 0.799308 | 0.063568 |
| 28 | 0.934061 | 0.025269 |
| 29 | 0.865279 | 0.061029 |
| 30 | 0.842996 | 0.039019 |
| 31 | 0.817903 | 0.077422 |
| 32 | 0.877667 | 0.072959 |
| 33 | 0.761753 | 0.050388 |
| 34 | 0.894998 | 0.061223 |
| 35 | 0.725972 | 0.038464 |
| 36 | 0.713406 | 0.059993 |
| 37 | 0.820271 | 0.064705 |
| 38 | 0.89279 | 0.048846 |
| 39 | 0.104985 | 0.060424 |
| 40 | 0.305773 | 0.0925 |
| 41 | 0.353877 | 0.096278 |
| 42 | 0.332419 | 0.073805 |
| 43 | 0.302836 | 0.058051 |
| 44 | 0.269746 | 0.06962 |
| 45 | 0.285574 | 0.077665 |
| 46 | 0.281007 | 0.095874 |
| 47 | 0.349212 | 0.07144 |
| 48 | 0.25321 | 0.07674 |
| 49 | 0.370315 | 0.070673 |
| 50 | 0.174516 | 0.094198 |
| 51 | 0.258541 | 0.081338 |
| 52 | 0.140918 | 0.093864 |
| 53 | 0.442887 | 0.099052 |
| 54 | 0.31481 | 0.067329 |
| 55 | 0.133713 | 0.097183 |
| 56 | 0.391563 | 0.077072 |
| 57 | 0.272224 | 0.126331 |
| 58 | 0.291766 | 0.069656 |
| 59 | 0.200267 | 0.083512 |
| 60 | 0.074471 | 0.086488 |
| 61 | 0.34979 | 0.089014 |
| 62 | 0.216896 | 0.089778 |
| 63 | 0.381389 | 0.101821 |
| 64 | 0.279454 | 0.104565 |
| 65 | 0.153053 | 0.088728 |
| 66 | 0.338448 | 0.082237 |
