# Supplementary material for: Hypernetwork Construction and Feature Fusion Analysis Based on Sparse Group Lasso Method on fMRI Dataset
Source: Front Neurosci. 2020 Feb 12;14:60. doi: 10.3389/fnins.2020.00060 (PMC7029661; doi:10.3389/fnins.2020.00060)
Supplement: TABLE S4 — Classification performance of the three methods about AD dataset. [file Table_4.docx]

Supplemental Table S4**.** Classification performance of the three methods about AD dataset

| **Methods** | **Research** | **Accuracy** | **Sensitivity** | **Specificity** | **BAC** |
| --- | --- | --- | --- | --- | --- |
| **Lasso-based** | Multi-feature | 81.35% | 86.21% | 76.67% | 81.44% |
| **gLasso-based** | Multi-feature | 84.74% | 90.00% | 79.31% | 84.66% |
| **sgLasso-based** | Multi-feature | 88.13 % | 89.66% | 86.67% | 88.17% |
