# Supplementary material for: Hypernetwork Construction and Feature Fusion Analysis Based on Sparse Group Lasso Method on fMRI Dataset
Source: Front Neurosci. 2020 Feb 12;14:60. doi: 10.3389/fnins.2020.00060 (PMC7029661; doi:10.3389/fnins.2020.00060)
Supplement: TEXT S1 — Image Acquisition. [file Data_Sheet_1.docx]

**Supplemental Text S1. Image Acquisition**

All the subjects were carried out resting state functional MRI scan by 3T MR equipment (Siemens Trio 3-Tesla scanner，Siemens, Erlangen, Germany). During the scan, subjects were requested to relax and their eyes closed, but not to fall asleep. Subjects wore spongy ear plugs and was placed carefully in the coil and provided cozy support.

248 contiguous EPI functional volumes was included in each scan(33 axial slices, repetition time (TR) = 2000 ms, echo time (TE) = 30 ms, thickness/skip = 4/0 mm, field of view (FOV) = 192×192 mm, matrix = 64×64 mm, flip angle = 90°) and the first ten volumes of time series were abandoned because of magnetization stabilization.
