# Supplementary material for: Hypernetwork Construction and Feature Fusion Analysis Based on Sparse Group Lasso Method on fMRI Dataset
Source: Front Neurosci. 2020 Feb 12;14:60. doi: 10.3389/fnins.2020.00060 (PMC7029661; doi:10.3389/fnins.2020.00060)
Supplement: TEXT S2 — Results of multiple linear regression analysis between network properties and confounding variables (cluster coefficients based on single node). [file Data_Sheet_2.docx]

**Supplemental Text S2. Results of multiple linear regression analysis between network properties and confounding variables.**

| Confounding Variables | Coefficients | Std. Error | T Stat. | P-value | Lower 95% | Upper 95% |
| --- | --- | --- | --- | --- | --- | --- |
| lasso | | | | | | |
| Clustering Coefficient HCC^1^ (Adj. R_sqr_ = -0.037_,_ P = 0.878) | | | | | | |
| Intercept | 1.3082 | 0.0468 | 28.4128 | <0.0010 | 1.2165 | 1.4001 |
| Gender | -0.0100 | 0.0171 | -0.582 | 0.5634 | -0.0454 | 0.0256 |
| Age | 0.0012 | 0.0014 | 0.6512 | 0.5178 | -0.0012 | 0.0024 |
| Educational Attainments | 0.0000 | 0.0073 | 0.0523 | 0.9586 | -0.0128 | 0.0138 |
| Clustering Coefficient HCC^2^ (Adj. R_sqr_ = 0.021_,_ P = 0.230) | | | | | | |
| Intercept | 0.8386 | 0.1067 | 7.9291 | <0.0010 | 0.6275 | 1.0494 |
| Gender | 0.0611 | 0.0407 | 1.5163 | 0.1342 | -0.0197 | 0.1416 |
| Age | 0.0012 | 0.0028 | 0.4617 | 0.6464 | -0.0031 | 0.0056 |
| Educational Attainments | 0.0163 | 0.0151 | 1.0666 | 0.2914 | -0.0145 | 0.0462 |
| Clustering Coefficient HCC^3^ (Adj. R_sqr_ = -0.003_,_ P = 0.434) | | | | | | |
| Intercept | 0.3107 | 0.0574 | 5.4392 | <0.0010 | 0.1961 | 0.4245 |
| Gender | 0.0280 | 0.0224 | 1.2960 | 0.2000 | -0.0153 | 0.0711 |
| Age | -5.625E^-5^ | 0.0014 | -0.0515 | 0.9601 | -0.0026 | 0.0024 |
| Educational Attainments | 0.0074 | 0.0080 | 0.8172 | 0.4175 | -0.0104 | 0.0235 |
| group_lasso | | | | | | |
| Clustering Coefficient HCC^1^ (Adj. R_sqr_ = 0.062_,_ P = 0.174) | | | | | | |
| Intercept | 1.6268 | 0.0774 | 21.0141 | <0.0010 | 1.4716 | 1.7801 |
| Gender | -0.0142 | 0.0293 | -0.4787 | 0.6354 | -0.0735 | 0.0453 |
| Age | -0.0044 | 0.0021 | -1.6828 | 0.1166 | -0.0072 | 0.0013 |
| Educational Attainments | -0.0082 | 0.0116 | -0.7382 | 0.4634 | -0.0304 | 0.0143 |
| Clustering Coefficient HCC^2^ (Adj. R_sqr_ = 0.146_,_ P = 0.205) | | | | | | |
| Intercept | 1.2345 | 0.1112 | 11.1140 | <0.0010 | 1.0124 | 1.4566 |
| Gender | 0.0071 | 0.0428 | 0.1661 | 0.8698 | -0.0772 | 0.0916 |
| Age | 0.0088 | 0.0025 | 1.3926 | 0.1244 | 0.0041 | 0.0121 |
| Educational Attainments | 0.0011 | 0.0163 | 0.0395 | 0.9695 | -0.0304 | 0.0326 |
| Clustering Coefficient HCC^3^ (Adj. R_sqr_ = -0.009_,_ P = 0.492) | | | | | | |
| Intercept | 0.4282 | 0.0877 | 4.9391 | <0.0010 | 0.2554 | 0.6012 |
| Gender | 0.0397 | 0.0336 | 1.1883 | 0.2392 | -0.0275 | 0.1053 |
| Age | 2.366E^-6^ | 0.0024 | 0.0013 | 0.9994 | -0.0034 | 0.0030 |
| Educational Attainments | 0.0100 | 0.0124 | 0.7994 | 0.4273 | -0.0151 | 0.0344 |
| Sparse group lasso | | | | | | |
| Clustering Coefficient HCC^1^ (Adj. R_sqr_ = -0.0296_,_ P = 0.5914) | | | | | | |
| Intercept | 1.5296 | 0.1190 | 12.8578 | <0.0010 | 1.2879 | 1.7714 |
| Gender | -0.0266 | 0.0476 | -0.5578 | 0.5806 | -0.1234 | 0.0702 |
| Age | -0.0024 | 0.0023 | -1.0553 | 0.2987 | -0.0071 | 0.0022 |
| Educational Attainments | -0.0094 | 0.0167 | -0.5642 | 0.5763 | -0.0434 | 0.0245 |
| Clustering Coefficient HCC^2^ (Adj. R_sqr_ = -0.0749_,_ P = 0.9349) | | | | | | |
| Intercept | 1.5440 | 0.1834 | 8.4171 | <0.0010 | 1.1712 | 1.9168 |
| Gender | -0.0212 | 0.0734 | -0.2886 | 0.7746 | -0.1705 | 0.1281 |
| Age | -0.0016 | 0.0035 | -0.4558 | 0.6514 | -0.0088 | 0.0056 |
| Educational Attainments | -0.0073 | 0.0258 | -0.2831 | 0.7788 | -0.0597 | 0.0451 |
| Clustering Coefficient HCC^3^ (Adj. R_sqr_ = -0.0176_,_ P = 0.5096) | | | | | | |
| Intercept | 0.8625 | 0.1761 | 4.8972 | <0.0010 | 0.5046 | 1.2204 |
| Gender | -0.0524 | 0.0705 | -0.7425 | 0.4629 | -0.1957 | 0.0910 |
| Age | -0.0029 | 0.0034 | -0.8408 | 0.4064 | -0.0097 | 0.0040 |
| Educational Attainments | -0.0210 | 0.0247 | -0.8492 | 0.4017 | -0.0713 | 0.0293 |

The range of age is 17–51 years. Optional values of gender are male and female. Optional values of educational attainments are illiteracy, primary school, junior high school, senior high school, junior college, college, graduate degree and above. Adj. R_sqr_, adjusted R square.Coefficients, regression coefficient. Std. Error, standard error. T stat., T statistic. Lower 95%, low bound of 95% confidence limits. Upper 95%, upper bound of 95% confidence limits.
