# Supplementary material for: Hypernetwork Construction and Feature Fusion Analysis Based on Sparse Group Lasso Method on fMRI Dataset
Source: Front Neurosci. 2020 Feb 12;14:60. doi: 10.3389/fnins.2020.00060 (PMC7029661; doi:10.3389/fnins.2020.00060)
Supplement: TEXT S3 — Results of multiple linear regression analysis between network properties and confounding variables (cluster coefficients based on a pair of nodes). [file Data_Sheet_3.docx]

**Supplemental Text S3.** **Results of multiple linear regression analysis between network properties and confounding variables.**

| Confounding Variables | Coefficients | Std. Error | T Stat. | P-value | Lower 95% | Upper 95% |
| --- | --- | --- | --- | --- | --- | --- |
| Lasso | | | | | | |
| Clustering Coefficient COMHCC^1^ (Adj. R_sqr_ = 0.009859_,_ P = 0.3553) | | | | | | |
| Intercept | 0.0509 | 0.0034 | 15.1947 | <0.0010 | 0.0442 | 0.0578 |
| Gender | 0.0010 | 0.0013 | 0.81778 | 0.4193 | -0.0016 | 0.0036 |
| Age | -3.4E-05 | 6.21E-05 | -0.5528 | 0.5841 | -0.0002 | 9.2E-05 |
| Educational Attainments | -0.0008 | 0.0005 | -1.7850 | 0.0835 | -0.0017 | 0.0001 |
| Clustering Coefficient COMHCC^2^ (Adj. R_sqr_ = -0.0534_,_ P = 0.7714) | | | | | | |
| Intercept | 0.0639 | 0.0048 | 13.2976 | <0.0010 | 0.0542 | 0.0737 |
| Gender | 0.0013 | 0.0019 | 0.7076 | 0.4840 | -0.0026 | 0.0053 |
| Age | 1.74E-05 | 9.25E-05 | 0.1881 | 0.8519 | -0.0001 | 0.0002 |
| Educational Attainments | -0.0006 | 0.0007 | -0.8454 | 0.4038 | -0.0020 | 0.0008 |
| Clustering Coefficient COMHCC^3^ (Adj. R_sqr_ = -0.0105_,_ P = 0.3502) | | | | | | |
| Intercept | 0.3192 | 0.0405 | 7.8787 | <0.0010 | 0.2368 | 0.4015 |
| Gender | -0.0183 | 0.0162 | -1.1293 | 0.2667 | -0.0512 | 0.0146 |
| Age | -0.0008 | 0.0008 | -0.9683 | 0.3397 | -0.0023 | 0.0008 |
| Educational Attainments | -0.0042 | 0.0057 | -0.7350 | 0.4674 | -0.0158 | 0.0074 |
| Clustering Coefficient COMHCC^4^ (Adj. Rsqr = 0.0671, P = 0.1502) | | | | | | |
| Intercept | 0.1177 | 0.0042 | 27.7512 | <0.0010 | 0.1090 | 0.1263 |
| Gender | 0.0004 | 0.00170 | 0.23673 | 0.8143 | -0.0031 | 0.0039 |
| Age | -7.2E-05 | 8.15E-05 | -0.8874 | 0.3811 | -0.0002 | 9.33E-05 |
| Educational Attainments | -0.0014 | 0.0006 | -2.2942 | 0.0281 | -0.0026 | -0.0002 |
| Clustering Coefficient COMHCC^5^ (Adj. Rsqr = 0.0419, P = 0.2221) | | | | | | |
| Intercept | 1.6451 | 0.1352 | 12.1687 | <0.0010 | 1.3704 | 1.9199 |
| Gender | 0.0641 | 0.0541 | 1.1840 | 0.2446 | -0.0459 | 0.1741 |
| Age | -0.0006 | 0.0026 | -0.2481 | 0.8055 | -0.0059 | 0.0046 |
| Educational Attainments | -0.0387 | 0.0190 | -2.0373 | 0.0495 | -0.0773 | -9.6E-05 |
| Group lasso | | | | | | |
| Clustering Coefficient COMHCC^1^ (Adj. R_sqr_ = -0.004_,_ P = 0.4292) | | | | | | |
| Intercept | 0.0519 | 0.0068 | 7.6487 | <0.0010 | 0.0381 | 0.0657 |
| Gender | 0.0030 | 0.0027 | 1.0988 | 0.2796 | -0.0025 | 0.0085 |
| Age | 0.0001 | 0.0001 | 0.9056 | 0.3715 | -0.0002 | 0.0004 |
| Educational Attainments | 0.0005 | 0.0010 | 0.5333 | 0.5973 | -0.0014 | 0.0024 |
| Clustering Coefficient COMHCC^2^ (Adj. R_sqr_ = 0.016_,_ P = 0.3227) | | | | | | |
| Intercept | 0.0623 | 0.0089 | 6.9860 | <0.0010 | 0.0442 | 0.0804 |
| Gender | 0.0051 | 0.0036 | 1.4379 | 0.1596 | -0.0021 | 0.0124 |
| Age | 0.0001 | 0.0002 | 0.8763 | 0.3870 | -0.0002 | 0.0005 |
| Educational Attainments | 0.000444 | 0.001254 | 0.3541 | 0.7254 | -0.0021 | 0.0030 |
| Clustering Coefficient COMHCC^3^ (Adj. R_sqr_ = 0.1117_,_ P = 0.0719) | | | | | | |
| Intercept | 0.5248 | 0.0765 | 6.8617 | <0.0010 | 0.3694 | 0.6803 |
| Gender | -0.0597 | 0.0306 | -1.9509 | 0.0594 | -0.1220 | 0.0025 |
| Age | -0.0025 | 0.0015 | -1.6690 | 0.1043 | -0.0054 | 0.0005 |
| Educational Attainments | 0.0035 | 0.01075 | 0.3223 | 0.7492 | -0.0184 | 0.0253 |
| Clustering Coefficient COMHCC^4^ (Adj. R_sqr_ = -0.0810_,_ P = 0.9723) | | | | | | |
| Intercept | 0.1344 | 0.0072 | 18.7239 | <0.0010 | 0.1198 | 0.1490 |
| Gender | 0.0004 | 0.0029 | 0.14096 | 0.8887 | -0.0054 | 0.0062 |
| Age | 3.53E-05 | 0.0001 | 0.25570 | 0.7997 | -0.0003 | 0.0003 |
| Educational Attainments | 0.0004 | 0.0010 | 0.35023 | 0.7283 | -0.0017 | 0.0024 |
| Clustering Coefficient COMHCC^5^ (Adj. R_sqr_ = -0.0577, P = 0.8059) | | | | | | |
| Intercept | 3.3837 | 0.7335 | 4.6127 | <0.0010 | 1.8929 | 4.8744 |
| Gender | -0.1454 | 0.2937 | -0.4949 | 0.6239 | -0.7422 | 0.4515 |
| Age | 0.0018 | 0.0141 | 0.1308 | 0.8967 | -0.0268 | 0.0305 |
| Educational Attainments | 0.0987 | 0.1031 | 0.9571 | 0.3452 | -0.1108 | 0.3081 |
| Sparse group lasso | | | | | | |
| Clustering Coefficient COMHCC^1^ (Adj. R_sqr_ = -0.0405_,_ P = 0.6715) | | | | | | |
| Intercept | 0.0615 | 0.0138 | 4.4714 | <0.0010 | 0.0336 | 0.0895 |
| Gender | 0.0062 | 0.0055 | 1.1325 | 0.2653 | -0.0050 | 0.0174 |
| Age | 9.21E-05 | 0.0003 | 0.3479 | 0.7301 | -0.0005 | 0.0006 |
| Educational Attainments | -0.0002 | 0.0019 | -0.1072 | 0.9152 | -0.0041 | 0.0037 |
| Clustering Coefficient COMHCC^2^ (Adj. R_sqr_ = -0.0225_,_ P = 0.5417) | | | | | | |
| Intercept | 0.0686 | 0.0173 | 3.9781 | <0.0010 | 0.0335 | 0.1037 |
| Gender | 0.0086 | 0.0069 | 1.2496 | 0.2200 | -0.0054 | 0.0227 |
| Age | 0.0002 | 0.0003 | 0.5426 | 0.5910 | -0.0004 | 0.0009 |
| Educational Attainments | 0.0002 | 0.0024 | 0.0776 | 0.9386 | -0.0047 | 0.0051 |
| Clustering Coefficient COMHCC^3^ (Adj. R_sqr_ = 0.0431_,_ P = 0.2118) | | | | | | |
| Intercept | 0.5431 | 0.0770 | 7.0561 | <0.0010 | 0.3867 | 0.6995 |
| Gender | -0.0537 | 0.0308 | -1.7418 | 0.0906 | -0.1163 | 0.0090 |
| Age | -0.0009 | 0.0015 | -0.6158 | 0.5421 | -0.0039 | 0.0021 |
| Educational Attainments | -0.0060 | 0.0108 | -0.5518 | 0.5847 | -0.0280 | 0.0160 |
| Clustering Coefficient COMHCC^4^ (Adj. R_sqr_ = -0.0679_,_ P = 0.8849) | | | | | | |
| Intercept | 0.1523 | 0.0147 | 10.3911 | <0.0010 | 0.1225 | 0.1821 |
| Gender | 0.0036 | 0.0059 | 0.6178 | 0.5408 | -0.0083 | 0.0155 |
| Age | 8.28E-05 | 0.0003 | 0.2938 | 0.7707 | -0.0005 | 0.0007 |
| Educational Attainments | -0.0009 | 0.0021 | -0.4342 | 0.6669 | -0.0051 | 0.0033 |
| Clustering Coefficient COMHCC^5^ (Adj. R_sqr_ = -0.0847_,_ P = 0.9901) | | | | | | |
| Intercept | 3.9741 | 0.9129 | 4.3530 | <0.0010 | 2.1188 | 5.8294 |
| Gender | 0.1061 | 0.3655 | 0.2903 | 0.7734 | -0.6368 | 0.8489 |
| Age | -0.001 | 0.0176 | -0.0568 | 0.9551 | -0.0367 | 0.0347 |
| Educational Attainments | -0.0310 | 0.1283 | -0.2413 | 0.8108 | -0.2917 | 0.2298 |

The range of age is 17–51 years. Optional values of gender are male and female. Optional values of educational attainments are illiteracy, primary school, junior high school, senior high school, junior college, college, graduate degree and above. Adj. R_sqr_, adjusted R square.Coefficients, regression coefficient. Std. Error, standard error. T stat., T statistic. Lower 95%, low bound of 95% confidence limits. Upper 95%, upper bound of 95% confidence limits.
