# Supplementary material for: Hypernetwork Construction and Feature Fusion Analysis Based on Sparse Group Lasso Method on fMRI Dataset
Source: Front Neurosci. 2020 Feb 12;14:60. doi: 10.3389/fnins.2020.00060 (PMC7029661; doi:10.3389/fnins.2020.00060)
Supplement: TEXT S4 — Power analysis for evaluating if the samples size was enough. [file Data_Sheet_4.docx]

**Supplemental Text S4. Power analysis for evaluating if the samples size was enough**

Power analysis can help determine the sample size required for a given effect size when degree of confidence is fixed, which involve four quantities: sample size, significance level, power, and effect size. In this study, according to the general situation, the significance level and efficacy were set as 5% and 90%, respectively. Adjusted R-squared value of regression analysis was regard as the effect size, where regression analysis was carried out finding the correlation of the brain regions between MDD group and the NC group, in other words, the mean cluster coefficient of each group of (NC and MDD) subjects was performed by regression analysis. The effect value is: lasso (HCC: 0.920; COMHCC: 0.810); gLasso (HCC: 0.820; COMHCC: 0.835); sgLasso (HCC: 0.845; COMHCC: 0.865). Power analysis was performed according to the two-sample t-test, and the required sample sizes finally obtained were lasso (HCC: 25; COMHCC: 33); gLasso (HCC: 32; COMHCC: 31); sgLasso (HCC: 30; COMHCC: 29). The results show that the size of the required sample is enough as the size of the depression data set used in our experiments.

But it cannot be ignored that a common problem in this type of disease research is that there are fewer data sets. The reason is the difficulties of sample collection, the number of subjects often has failed to provide the desired scale in similar studies, especially those when the subjects are patients. A review was conducted of 76 recent studies on machine learning related to brain networks (Supplemental Material Table S3). Some of these studies were cited in a 2016 review of the literature on machine learning and brain networks [1] and a 2016 review of the literature on depression and machine learning [2]. Additional studies published in the past four years (2016–2018) were included. The review indicated that there were 39 (51.31%) studies that selected less than 38 subjects as the number of samples. This result showed that small sample size is applied into most similar researches and even with small sample sizes, studies in this area can produce significant findings.

[1] BROWN C J and HAMARNEH G, Machine Learning on Human Connectome Data from MRI[J]*.* 2016.

[2] PATEL M J, KHALAF A, and AIZENSTEIN H J, Studying depression using imaging and machine learning methods[J]*.* Neuroimage Clinical, 2016. 10(4): 115-123.
